# Renal hypoxia–HIF–PHD–EPO signaling in transition metal nephrotoxicity: friend or foe?

**DOI:** 10.1007/s00204-022-03285-3

**Published:** 2022-04-21

**Authors:** Frank Thévenod, Timm Schreiber, Wing-Kee Lee

**Affiliations:** 1 Institute for Physiology, Pathophysiology and Toxicology, ZBAF, Witten/Herdecke University, Stockumer Strasse 12, 58453 Witten, Germany; 2 Physiology and Pathophysiology of Cells and Membranes, Medical School EWL, Bielefeld University, R.1 B2-13, Morgenbreede 1, 33615 Bielefeld, Germany

**Keywords:** Anemia, Kidney, Metal toxicology, Oxidative stress, ROS, von Hippel-Lindau

## Abstract

The kidney is the main organ that senses changes in systemic oxygen tension, but it is also the key detoxification, transit and excretion site of transition metals (TMs). Pivotal to oxygen sensing are prolyl-hydroxylases (PHDs), which hydroxylate specific residues in hypoxia-inducible factors (HIFs), key transcription factors that orchestrate responses to hypoxia, such as induction of erythropoietin (EPO). The essential TM ion Fe is a key component and regulator of the hypoxia–PHD–HIF–EPO (HPHE) signaling axis, which governs erythropoiesis, angiogenesis, anaerobic metabolism, adaptation, survival and proliferation, and hence cell and body homeostasis. However, inadequate concentrations of essential TMs or entry of non-essential TMs in organisms cause toxicity and disrupt health. Non-essential TMs are toxic because they enter cells and displace essential TMs by ionic and molecular mimicry, e. g. in metalloproteins. Here, we review the molecular mechanisms of HPHE interactions with TMs (Fe, Co, Ni, Cd, Cr, and Pt) as well as their implications in renal physiology, pathophysiology and toxicology. Some TMs, such as Fe and Co, may activate renal HPHE signaling, which may be beneficial under some circumstances, for example, by mitigating renal injuries from other causes, but may also promote pathologies, such as renal cancer development and metastasis. Yet some other TMs appear to disrupt renal HPHE signaling, contributing to the complex picture of TM (nephro-)toxicity. Strikingly, despite a wealth of literature on the topic, current knowledge lacks a deeper molecular understanding of TM interaction with HPHE signaling, in particular in the kidney. This precludes rationale preventive and therapeutic approaches to TM nephrotoxicity, although recently activators of HPHE signaling have become available for therapy.

## Introduction

Interactions between the environment and organisms are crucial for life. Careless and widespread use of pesticides, plastics, chemicals, and environmental pollutants in general, is one of the most serious problems affecting human health in the twenty-first century (Klaassen 2019). Metals and metal compounds disrupt the function of various organs, such as the central nervous system (CNS), the hematopoietic system, the liver, and the kidneys (Ufelle and Barchowsky 2019).The kidneys are complex organs that are vital for the maintenance of normal body homeostasis. A kidney contains over 1 million functional units called nephrons, each composed of a glomerulus and a tubule. Ultrafiltration of the blood occurs at the glomerulus, forming a primary filtrate that is free of cells and large proteins and enters the tubular lumen. Renal tubules are highly specialized in their various segments, producing the final urine through reabsorption and secretion of filtered solutes and water. Filtration, reabsorption, and secretion processes maintain homeostatic levels of water, minerals, electrolytes, and hydrogen ions as well as eliminating metabolic waste products and xenobiotics from the body (Yu et al. 2019). Moreover, the kidneys are important endocrine organs. They secrete humoral factors that regulate blood pressure (renin), blood calcium concentration (calcitriol) and red blood cell production (erythropoietin; EPO) (reviewed in Kurt and Kurtz 2015).

### Renal handling of transition metals

The kidney is also a target organ of metal toxicity for its capability to filter, reabsorb, excrete and accumulate metal ions. Although, it has been assumed for a long time that the kidney plays no part in metal homeostasis, it is now established that the kidney is involved in transport of iron and other metal ions (Smith and Thévenod 2009; Thévenod and Wolff 2016; van Swelm et al. 2020). Several metal transport proteins have been identified in the kidney, including the multi-ligand receptor complex megalin:cubilin:amnionless (Christensen and Birn 2002) or the divalent metal transporter-1 (DMT-1; gene *SLC11A2*) that transports ferrous iron and a broad range of other divalent transition metals (TMs), such as cadmium, zinc, manganese, cobalt and nickel (Gunshin et al. 1997; Illing et al. 2012). Some TM ions are used as cofactors in enzymatic reactions, as they can readily transit between oxidized and reduced states, e.g. for nucleic acid and protein synthesis, enzymatic reactions, membrane stabilization, immune system, antioxidant defense, oxidative phosphorylation, etc. (Fraga 2005; Zoroddu et al. 2019), making them essential for cellular homeostasis.

One of the best studied examples of essential TM function is the role of iron in erythropoiesis in red bone marrow (Ganz 2013). Iron is required in sufficient amounts in erythroblasts where it is used for hemoglobin synthesis to maintain O_2_ transporting capacity and to prevent anemia. Iron is transported in the blood by transferrin (TF) and is then released to erythroblasts by interaction of di-ferric TF with the TF receptor (TFR). The TF-TFR endosomal cycle is indispensable for erythropoiesis, as erythroblasts do not have an alternative route for iron import. Additionally, iron affects erythropoiesis by contributing to the regulation of EPO production in the kidney through coordinated function of the iron-responsive element-binding protein 1 (IRP1) and hypoxia-inducible factors (HIFs). IRP1 binds to the iron-responsive element (IRE) present in several genes (Hentze et al. 2010).

In general, essential TMs are effective at very low concentrations, demanding tight regulation. Both deficiency and excess may cause severe illness or death (Bleackley and Macgillivray 2011; Crichton 2017). Transport and urinary excretion of essential metal ions by the kidney, together with the gastrointestinal absorption rates, contribute to keeping their plasma concentration low to prevent their accumulation in tissues and cells, which may result in organ damage and dysfunction, e.g. by causing cell death, inflammation and cancer. Non-essential metal ions, such as cadmium, lead and mercury serve no known purpose in the human body, but similar to essential metal ions, they are transported and excreted by the kidney and use the same transport pathways, hence they may accumulate and induce nephrotoxicity (Bridges and Zalups 2017; Satarug et al. 2020). When TMs, essential or non-essential, enter the body, the kidney is affected by uptake of the toxic metals into renal cells, which in turn leads to concomitant decrease of essential metal entry due to competition between the metal ions. The severity of renal damage depends on the metal concentration and duration of exposure. The sequels of acute intoxication will differ from those induced by chronic intoxication. Certain metals are known to generate free radicals either by their own redox activity or by interfering with reactive oxygen species (ROS) scavenging mechanisms, which then may lead to oxidative stress and cause cellular damage, resulting finally in cell death (Valko et al. 2005). Moreover, some metals are known to have carcinogenic effects. Several signaling proteins or cellular regulatory proteins that participate in apoptosis, cell cycle regulation, DNA repair, DNA methylation, cell growth, and differentiation are targets of metals (Chen et al. 2019a; Tokar et al. 2011). In recent years, mounting evidence from individual reports has revealed that TM exposure increases the risk of anemia (e.g. Ashley-Martin et al. 2021; Bayhan et al. 2017; Choi et al. 2017; Lopez-Rodriguez et al. 2017; Shen et al. 2019; Yadav et al. 2020).

### The kidney as a sensor of hypoxia

Hypoxia is a condition in which a cell or an organ has insufficient oxygen (O_2_) supply. Stimulation of red blood cell production is one of the classical physiological responses to systemic hypoxia during anemia. In the kidneys, branches of renal arteries and veins run in parallel over long distances in close contact with each other. This special architecture allows O_2_ to diffuse from the arterial system into the venous system before reaching the capillary bed (“arteriovenous O_2_ shunting”) and leads to relatively low O_2_ tensions in kidney tissues, despite high blood flow to the kidney (20% of cardiac output) (reviewed in Eckardt et al. 2005; Evans et al. 2008). O_2_ tension in the renal cortex is around 30–50 mmHg and does not rise above 10–25 mmHg in the renal medulla (Nangaku and Eckardt 2007) in comparison with blood (up to 100 mmHg) or well-oxygenated tissues like the intestine (up to 71 mmHg) (Carreau et al. 2011). Due to their high metabolic activity, renal tubules display high O_2_ consumption. Consequently, low O_2_ supply and high O_2_ demand make the kidneys particularly sensitive to changes in O_2_ delivery. When the kidneys sense hypoxia, peritubular fibroblasts-like interstitial cells (termed renal EPO-producing cells, REPCs) start to produce EPO. However, in rodents hypoxia-responsive *Epo* expression is limited to a small number of these fibroblasts in the cortico-medullary junction, that is the deep renal cortex (predominantly juxtamedullary region) and outer medulla (Bachmann et al. 1993; Eckardt et al. 1993; Koury et al. 1989; Maxwell et al. 1993; Paliege et al. 2010) (see Table 1 for a synopsis of EPO expression in renal structures). Although basal *Epo* expression was also seen in rodent tubular cells, these cells show almost no response to hypoxia (Nagai et al. 2014).The increased expression of rat *Epo* in the context of anemia involves progressive recruitment of additional REPCs situated more superficially in the kidney cortex (Eckardt et al. 1993). The origin of these REPCs is still under debate. REPCs have a unique morphology as they have dendrite-like processes and express the PDGF receptor beta (PDGFB) and ecto-5′-nucleotidase (CD73) (Asada et al. 2011; Bachmann et al. 1993). A number of studies, using genetic cell fate technologies, indicate that REPCs may originate from a distinct *forkhead box protein D1* (*Foxd1*)-expressing subpopulation of migrating neural crest cells (Asada et al. 2011; Kobayashi et al. 2016; Souma et al. 2016; Yamazaki et al. 2013). The cell type-specific *EPO* gene expression may involve a GATA factor-binding motif (GATA box) that has been identified in the core promotor region of the *EPO* gene and acts as a negative regulatory element (Imagawa et al. 2002). This GATA-based repression seems to contribute to the switch of EPO production from liver to kidney during development (Dame et al. 2004), and may prevent EPO expression in epithelial cells, including nephron epithelia, despite hypoxic conditions (Kaneko et al. 2017; Obara et al. 2008). In other cell types or organs, EPO expression may be permanently silenced by epigenetic mechanisms, however, the exact mechanisms of organ and cell type-specific EPO production remain to be elucidated.

**Table 1:** Expression of HIFs, PHDs and EPO in rodent renal structures

Renal structure	HIF isoform	HIF stabilization				PHD		EPO expression	References
		Carbon monoxide	Cobaltous chloride	Ischemia	Hypoxia	mRNA	Protein		
Glomerulus	HIF1A HIF2A	− ++	− ++	+ +	+ +	*Egln2*+ *Egln1*+ *Egln3*+	PHD1++ PHD2− PHD3++	n.d	Bernhardt et al. (2006a); Rosenberger et al. (2002); Schodel et al. (2009)
Proximal tubule	HIF1A HIF2A	++ −	− −	− −	n.d n.d	*Egln2*+ *Egln1*++ *Egln3*+	PHD1− PHD2− PHD3−	Basal *Epo* expression	Bernhardt et al. (2006a); Nagai et al. (2014); Rosenberger et al. (2002); Schodel et al. (2009)
Thick ascending limb	HIF1A HIF2A	+ −	− −	− −	n.d n.d	*Egln2*++ *Egln1*++ *Egln3*++	PHD1++ PHD2++ PHD3++	n.d	Bernhardt et al. (2006a); Rosenberger et al. (2002); Schodel et al. (2009)
Distal convoluted tubule	HIF1A HIF2A	− −	++ −	− −	n.d n.d	*Egln2*+ *Egln1*++ *Egln3*+	PHD1++ PHD2++ PHD3++	Basal *Epo* expression	Bernhardt et al. (2006a); Nagai et al. (2014); Rosenberger et al. (2002); Schodel et al. (2009)
Collecting ducts	HIF1A HIF2A	++ −	++ −	++ −	+ −	*Egln2*+ *Egln1*+ *Egln3*+	PHD1++ PHD2++ PHD3++	Basal *Epo* expression	Bernhardt et al. (2006a); Nagai et al. (2014); Rosenberger et al. (2002); Schodel et al. (2009)
Endothelial cells	HIF1A HIF2A	− +	− +	− +	n.d n.d	*Egln2* n.d *Egln1* n.d *Egln3* n.d	PHD1+ PHD2− PHD3+	n.d	Bernhardt et al. (2006a); Rosenberger et al. (2002); Schodel et al. (2009)
Interstitial fibroblasts	HIF1A HIF2A	+ +++	+ +	− +	− +	*Egln2* n.d *Egln1* n.d *Egln3* n.d	PHD1++ PHD2− PHD3++	Hypoxia-induced *Epo* expression	Bachmann et al. (1993); Eckardt et al. (1993); Koury et al. (1989); Maxwell et al. (1993); Nagai et al. (2014); Paliege et al. (2010); Rosenberger et al. (2002); Schodel et al. (2009)

*+* moderate expression, *++* strong expression, *−* no signal, *n.d* not determined

In renal disease, the hypoxic induction of EPO fails and anemia becomes more severe as the disease progresses without concomitant rise in EPO production (Erslev 1991). The main determinant of EPO production is the transcriptional activity of its gene, which is driven by O_2_ tension. Key mediators of this cellular adaptation to hypoxia are hypoxia-inducible factors (HIFs) (see “HIFs/PHDs: isoforms, regulation, tissue specific expression, mechanisms in the kidney”). Beside their role in EPO regulation, HIFs have different effects in the kidney. Chronic HIF activation may impair differentiation of renal progenitor cells, promote, or restrict cyst growth, and protect renal tubules in acute or chronic kidney injury (see “Impact of toxic metal ions on the renal HPHE signaling axis” and “Therapy of TM nephrotoxicity, controversies, outlook, and conclusions”). Furthermore, HIFs are involved in renal inflammation and fibrosis (for review see Schodel and Ratcliffe 2019). Whether TMs directly interfere with the renal hypoxia–PHD–HIF–EPO (HPHE) signaling axis remains unclear. Hence, in this review, we address the roles of O_2_ sensing and the HPHE signaling axis in the context of TM nephrotoxicity.

## HIFs/PHDs: isoforms, regulation, tissue-specific expression, mechanisms in the kidney

Cellular responses to hypoxia involve increased glycolysis to compensate for energy loss due to reduced oxidative phosphorylation, and at the systemic level, promotion of erythrocytosis and angiogenesis to achieve efficient O_2_ utilization. Key to this adaptation mechanism is the rapid accumulation of HIFs. HIF transcription factors are heterodimers of an O_2_-regulated alpha-subunit (HIF-alpha) and a constitutively expressed beta-subunit (HIF1B, also known as aryl hydrocarbon receptor nuclear translocator, ARNT) (Wang and Semenza 1995). In humans, the HIF-alpha subunits consist of three isoforms, HIF1A, HIF2A (also known as Endothelial PAS Domain-Containing Protein 1; gene *EPAS1*), and HIF3A (Ema et al. 1997; Gu et al. 1998), and when complexed with HIF1B they are named HIF1, HIF2 and HIF3. All HIF subunits are members of the basic helix-loop-helix PER-ARNT-SIM (PAS) protein family (Brahimi-Horn et al. 2005). Whereas HIF1A and HIF2A are well studied, little is known regarding the biological functions of HIF3A. *HIF3A* shows complex cellular expression patterns with multiple splicing forms, several of these isoforms lack the transactivation domain found in the C-termini of* HIF1A* and *HIF2A*, suggesting a different role as a negative regulator of hypoxia-inducible gene expression (Duan 2016; Hara et al. 2001). Nevertheless, some HIF3 target genes have been identified and it was shown that HIF3A contributes to various diseases, such as idiopathic pulmonary fibrosis (Aquino-Galvez et al. 2019; Zhang et al. 2014). The HIF-alpha subunits are continuously transcribed and translated into protein and are maintained at low levels by O_2_-dependent hydroxylation on specific proline residues. Hydroxylated-alpha subunits are recognized by the von Hippel-Lindau (VHL) protein of the E3 ubiquitin ligase complex and are then rapidly degraded via the polyubiquitination/proteasomal pathway (Maxwell et al. 1999). Under normoxia, HIF-alpha proteins have a half-life of less than 5 min in human cell lines (Huang et al. 1996). As intracellular O_2_ concentration decreases, non-hydroxylated HIF-alpha accumulates and forms the functional transcription factor complexes HIF1 and HIF2 in the nucleus by heterodimerization with the HIF1B subunit.

### *Regulation of HIF-alpha levels by O*_*2*_*-sensing PHDs*

HIF1A/2A stability and abundance are regulated by prolyl-4-hydroxylases, known as prolyl-hydroxylase domain proteins (PHDs) (also known as Egl-9 Family Hypoxia Inducible Factors 1-3; genes *EGLN1-3*) that function as oxygen sensors. HIF-alpha contain a C- and an N-terminal O_2_-dependant degradation (ODD) domain and PHDs hydroxylate two specific proline residues (HIF1A: Pro_402_ and Pro_564_, HIF2A: Pro_405_ and Pro_531_) in the ODD domain of human HIF-alpha (Kaelin 2005). In rodents and humans three PHD isoforms have been identified, PHD1/EGLN2, PHD2/EGLN1 and PHD3/EGLN3 (Epstein et al. 2001; Ivan et al. 2001; Jaakkola et al. 2001). All three enzymes use oxygen and 2-oxoglutarate (2-OG) as co-substrates and ferrous iron (Fe^2+^) and ascorbate (AA) as cofactors. Catalytic Fe^2+^ is bound in a bi-dentate manner by a 2-histidine-1-aspartate triad of amino residues. In the catalytic cycle, oxidation of the prolyl residue in HIF-alpha is coupled to the oxidative decarboxylation of 2-OG in a redox cycle that involves the creation of a ferryl-oxo (Fe^IV^ = O) intermediate at the catalytic center. Binding of HIF weakens complexation of a water molecule to the iron, thereby opening a coordination site for oxygen binding. The reactive Fe^IV^ = O intermediate oxidizes HIF via a direct insertion into a C-H bond. Product dissociation completes the catalytic cycle (Loenarz and Schofield 2011). Replacing the 2-OG in PHDs by dimethyloxalylglycine (DMOG), which is intracellularly converted to N-oxalylglycine (a structural analog of 2-OG), results in normoxic accumulation of HIFs in human cells (Epstein et al. 2001). AA is required by PHDs for their full catalytic capacity, as it reduces the catalytic iron center following the oxidation that occurs during uncoupled catalytic cycles (Flashman et al. 2010). In vitro studies show that the binding affinity of PHD enzymes for Fe^2+^ and 2-OG is unusually strong compared to other 2-OG oxygenases, which may reflect the pivotal role of HIF hydroxylases in hypoxic signaling to ensure that in the presence of sufficient concentrations of Fe^2+^ and 2-OG the enzyme is maintained in a form ‘primed’ for catalysis (McNeill et al. 2005).

PHDs differ in their expression patterns, tissue distribution, subcellular localization, and their ability to hydroxylate HIF-alpha (see Table 1 for an overview of PHD expression in the kidney). *Egln2* mRNA is highly expressed in murine testis, moderately in liver, and in low quantities in the heart, brain, and kidney (Lieb et al. 2002). PHD1 is a constitutively expressed protein with a nuclear localization sequence (Metzen et al. 2003; Steinhoff et al. 2009; Yasumoto et al. 2009), yet heterologously expressed human PHD1 shows no response to hypoxia (Metzen et al. 2003). *Egln1* mRNA is highly expressed in the murine heart and testis, and moderately in the brain, liver, and kidney (Lieb et al. 2002). Human PHD2 protein is primarily localized in the cytosol but shuttles between the nucleus and cytoplasm (Steinhoff et al. 2009). The nuclear localization of PHDs indicates that they hydroxylate HIF-alpha proteins also in the nucleus (reviewed in Depping et al. 2015). PHD2 has the lowest O_2_ affinity among the PHDs and is the most active and most important O_2_ sensor (Berra et al. 2003). Human PHD3 is present in both the cytoplasmic and nuclear compartment (Metzen et al. 2003), and its mRNA is expressed highly in the heart and liver, and moderately in the brain and kidney of mice (Lieb et al. 2002). Although mRNA of all three *Egln* isoforms are expressed in the rodent kidney, *Egln1* is most abundant in tubular cells, whereas *Egln2* and *Egln3* are predominantly expressed in interstitial fibroblasts of the kidney (Schodel et al. 2009). All human PHD proteins appear to be abundant in tubular segments of the inner medulla, where O_2_ tension is low (Soilleux et al. 2005). In human cells lines, all three PHDs can contribute to the regulation of both HIF1A and HIF2A, although PHD1 and PHD3 are more active on HIF2A than on HIF1A, whereas PHD2 hydroxylates HIF1A more efficiently (Appelhoff et al. 2004). Consistent with this study, PHD2 deficiency in mouse liver and kidney leads to the accumulation of nuclear HIF1A but not HIF2A (Takeda et al. 2007). In contrast, PHD1/PHD3 double deficiency in mice led to hepatic accumulation of HIF2A but not HIF1A (Takeda et al. 2008). Selectivity among HIF substrates is mediated by sequences contained within the mobile loop in the PHD polypeptide (Chowdhury et al. 2016; Villar et al. 2007). Strikingly, accumulation of HIF1A by hypoxia or inhibitors leads to feedback upregulation of human or rodent PHD2 and PHD3, but not PHD1, which prevents further accumulation of HIF1A (reviewed in Fong and Takeda 2008).

In addition to PHDs, factor inhibiting HIF (FIH) is a vital O_2_-sensitive enzyme for HIF regulation. FIH is an O_2_-dependent dioxygenase that, similarly to PHDs, requires Fe^2+^ and 2-OG. Human FIH hydroxylates an asparagine residue (HIF1A: Asn_803_, HIF2A: Asn_847_) in the C-terminal transactivation domain and prevents binding of the co-activators p300 and CREB-binding protein (CBP) to HIFs, which is required for full transcriptional activity (Lando et al. 2002a, b). Human FIH protein remains active at lower O_2_ concentrations than PHDs and dominates HIF activation during exposure to lower *P*_O2_ range (Stolze et al. 2004). *FIH* is expressed in distal tubules and podocytes in the kidney (Schodel et al. 2010). Interestingly, in human cells FIH is less effective on HIF2A than on HIF1A (Bracken et al. 2006).

### Renal expression of HIF-alpha isoforms and regulation of HIF-target genes

Whereas HIF1A is ubiquitously expressed, HIF2A exhibits a more tissue-specific expression pattern. In human and rat kidney, HIF1A is the predominant isoform in tubular cells, whereas HIF2A is strongly expressed in interstitial cells, endothelial cells and the glomeruli, but mostly absent from tubular cells (Bernhardt et al. 2006b; Rosenberger et al. 2002) (see Table 1 for a summary of HIF expression in the kidney). Beside their different expression patterns, HIF1 and HIF2 transcription factors also differ in their transcriptional targets, activation kinetics and O_2_ dependency. In human cell lines, HIF1A rapidly accumulates during severe hypoxia (< 5% O_2_) and takes part in the initial adaptation process to this condition, but then declines to low levels after 24 h, whereas HIF2A accumulation occurs—in addition to severe hypoxia—under prolonged and less severe hypoxic conditions (Holmquist-Mengelbier et al. 2006; Wiesener et al. 1998). This difference in kinetics may be due to the specific action of a negative feedback loop via an *HIF1A* antisense transcript that negatively regulates human HIF1A, but not HIF2A (Rossignol et al. 2002).

When HIFs accumulate in the nucleus under hypoxia, they bind to hypoxia response elements (HRE) in the enhancer or promotor region of their target genes that contain the core sequence RCGTG (R = A or G), resulting in transcription (Wenger et al. 2005). HIF1 seems to predominantly regulate glycolytic genes, whereas HIF2 preferentially regulates erythropoiesis and angiogenesis via *EPO* and vascular endothelial growth factor A (*VEGFA*), respectively (Hu et al. 2006; Morita et al. 2003; Warnecke et al. 2004). HIF1 increases almost all enzymes in the glycolytic pathway, as well as the glucose transporters 1 and 3 (Chen et al. 2001; Wenger 2002). Without sufficient O_2_, cells need to switch to O_2_-independent glycolysis to meet their energy demand, as the O_2_-dependent tricarboxylic acid cycle (TCA) is no longer operative (Dang and Semenza 1999; Seagroves et al. 2001). As glycolysis only generates two molecules ATP from each glucose molecule instead of 34–38 ATP molecules that the TCA cycle provides, cells need to increase glucose uptake. More elaborate adaptive responses to hypoxia seem to depend on HIF2. Hence, in response to systemic hypoxia, murine HIF proteins binds to the HRE in the 3’ enhancer region of *Epo* resulting in the rapid production of EPO by interstitial fibroblast-like cells to promote erythropoiesis (Koury et al. 1989; Semenza and Wang 1992), and at least in mice conditional ablation of HIF2A in the murine kidney has established that hypoxic induction of EPO is completely dependent on HIF2, even under severe hypoxia, and not on HIF1 (Kapitsinou et al. 2010; see also Gruber et al. 2007; Rankin et al. 2007; Scortegagna et al. 2005). In addition, EPO can also protect against kidney injury by reducing apoptosis and inflammation, and increasing tubular cell proliferation (for review see Moore and Bellomo 2011). VEGFA plays a central role in angiogenesis by activating the receptor tyrosine kinases VEGFR-1, -2, and -3 (Forsythe et al. 1996; Shibuya 2013), which may be important in pathological angiogenesis, promoting tumor growth and metastasis. In the kidney, the function of the glomerulus is dependent on the special vasculature maintained by VEGFA, and dysregulation may lead to glomerulopathy and breakdown of the filtration barrier (Eremina et al. 2008, 2003; Keir et al. 2017).

Relevant to this review, hypoxic cancer tissue may trigger HPHE signaling with consequent activation of hypoxia-induced target genes, such as growth factors (including *VEGFA*, *PDGFB*, and *TGFA*) that can then bind to their respective receptors and induce angiogenesis and proliferation. HIF transcription factors also increase the expression of several genes that regulate glucose metabolism (such as *SLC2A1*, *LDHA* and *PDK1*), as well as *EPO*, all to promote cancer cell proliferation, survival, angiogenesis and metabolic reprogramming. Moreover, certain types of kidney cancer, in particular clear cell renal carcinoma, display mutations in genes associated with HPHE signaling, especially *VHL* (Huang 2003; Linehan and Ricketts 2013; Linehan and Rouault 2013; Linehan et al. 2019, 2010), resulting in a “pseudo-hypoxic state” accompanied by increased EPO (and its receptor) expression (reviewed in Morais et al. 2013).

### VHL in renal cancer

Renal cancers have become increasingly prevalent, accounting for 2.2% of new adult malignancies and 1.8% of deaths. From the 36 cancer types that were reported, kidney cancer ranked 16th and is the 9th most common cancer type in males (Sung et al. 2021). Clear cell renal cell carcinoma (ccRCC) is the most common type of renal cancer and whilst it has been long known that it is a metabolic disease, comprehensive integrated molecular scrutinization utilizing whole genome sequencing, whole exome sequencing, RNA sequencing, array-based gene expression, copy number and DNA methylation analyses have now genetically and molecularly defined ccRCC (Cancer Genome Atlas Research et al. 2013; Sato et al. 2013). Up to 28 genes were identified to be significantly mutated in ccRCC with *VHL*, *PBRM1*, *SETD2* and *BAP1* belonging to the most significant mutated genes in both studies. Intriguingly, *VHL* is a key affected gene, occurring in approximately 60% of ccRCC, yet rarely in other renal cancer subtypes, such as papillary or chromophobe (Sukosd et al. 2003). *VHL* is located on chromosome 3p, which was deleted in more than 90% of patient samples (Zbar et al. 1987). Other key mutated genes, including the tumor suppressor gene *PBRM1*, are also located on the short arm of chromosome 3, which is frequently targeted in other cancer types, such as lung cancer (Zabarovsky et al. 2002). This leads to the question as to why chromosome 3p is susceptible to chromosomal aberrations and why *VHL* mutations are especially prevalent in ccRCC.

Due to the frequent alterations in chromosome 3p, it would be plausible to hypothesize susceptible regions are targeted during carcinogenesis. Common fragility sites (CFS) are chromosomal regions wherein breaks and gaps are often found, perturbing DNA replication and leading to replication stress (Glover et al. 2017). The structure of CFS—they are particularly AT-rich—may contribute to their sensitivity to damage and designation as “hotspots” of genomic instability. Interestingly, one of the most fragile sites has been localized to FRA3B region on chromosome 3p14 and is the top fragile locus in lymphoblasts (Hosseini et al. 2013; Huebner and Croce 2001). Furthermore, the tumor suppressor gene *FHIT* is localized to the FRA3B region and synergizes with *VHL*. However, a role for FRA3B/*FHIT* in ccRCC has been debated in conflicting studies. The low occurrence of terminal deletions encompassing the FRA3B region in nonpapillary RCC (3 from 100) (Bugert et al. 1997) or normal *FHIT* transcripts in renal cancer cell lines (van den Berg et al. 1997) lead to the conclusion that FRA3B/*FHIT* is not involved in the development of nonpapillary RCC. In a later study, a continuous deletion of a region of chromosome 3p, harboring *VHL* and *FHIT*, was found in 96% of ccRCC (Sukosd et al. 2003), which is supported by a number of earlier studies that speculate or imply involvement of the FRA3B region or *FHIT* in RCC (Hadaczek et al. 1998; Shridhar et al. 1997; Yamakawa et al. 1992). Decisively, the latest molecular analyses do not report *FHIT* among the significantly mutated genes in ccRCC (Cancer Genome Atlas Research et al. 2013; Sato et al. 2013), suggesting it does not drive ccRCC progression.

As a major site of excretion, the kidney comes into contact with numerous potentially toxic substances. Some of these substances are removed into the urine, yet others are retained in the kidney where they accumulate and may elicit stress and adaptive responses, culminating in cell death or survival. Risk factors for renal cancer include cigarette smoking, workplace exposures, obesity and hypertension. Could the *VHL* gene be directly targeted by potential carcinogens? Indeed, *VHL* mutations in renal tumors have been reported in nitrosamine-exposed Wistar rats (Shiao et al. 1998), potassium bromate-exposed F344 rats (Shiao et al. 2002) and individuals occupationally exposed to the industrial solvent and carcinogen trichloroethylene (Brauch et al. 2004). Furthermore, the impact of toxic substances and carcinogens on DNA damage (e.g. through oxidative stress), DNA replication, replication stress and DNA repair systems must be taken into consideration in the mutation of *VHL* and other tumor suppressor genes. To this end, transition metals are particularly relevant because of their ability to cause oxidative stress as well as their requirement for some enzymatic processes, such as DNA repair enzymes. Despite some indication of metals affecting HIFs and HIF-target genes (Li et al. 2006), the effect of transition metals on *VHL* mutation, expression and/or activity has remain largely unexplored.

## Transition metals: environmental presence, exposure, general modes of toxicity in the kidney

According to the International Union of Pure and Applied Chemistry (IUPAC), a TM is defined as "an element whose atom has an incomplete *d* sub-shell, or which can give rise to cations with an incomplete *d* sub-shell" (Chemistry IUoPaA 1997). Most TMs are found in the so-called *d*-block in the periodic table in groups 3–12 and have common features, such as electricity and heat conduction, malleability and, of particular importance from a biological point of view, multiple oxidation states, which is a result of their valence electrons (used to combine with other elements) being present in more than one shell. TMs naturally occur in the Earth, usually as salts, and their distribution was determined by geological events, such as geotectonic/metamorphic, volcanic and oceanic, that occurred. From the 38 elements found in the *d*-block, we have selected to review TMs that pose a potential threat to human health, namely, cadmium, chromium, cobalt, iron, nickel, and platinum. Of these, cobalt, iron and chromium are essential trace elements that are required for chemical reactions and physiological processes in the human body. The TMs copper and mercury have not been included because of little evidence and reports in the literature in the context of hypoxia and HIFs. The abundance of the selected TMs in the Earth’s crust follows the sequence: iron (Fe) > chromium (Cr) > nickel (Ni) > cobalt (Co) > cadmium (Cd) > platinum (Pt) (Haynes et al. 2016). Furthermore, Cd is in the top ten of the Agency for Toxic Substances and Disease Registry (ATSDR) 2019 Substance Priority List, which is based on their frequency, toxicity and potential for human exposure (ATSDR 2019).

TMs are released into routes of human exposure either by natural weathering or following industrial activities that mine and extract ores for further processing (Smith and Huyck 1999). Both methods would result in excess levels of TMs entering the food chain and further contributing to human exposure to the general population not employed in the industries. For both industrial workers and the general population, the inhalational and ingestion routes are the major entry pathways of TMs into the body; though the skin is penetrated by some metals, it is unlikely to be in large quantities. Maladies of the lung, intestinal tract, liver and kidney are usually reported. For the general nonsmoking population, exposure to contaminated water and foodstuffs is the major entry route and metal accumulation may contribute to diseases of the digestive tract and detoxification organs. Airborne pollutants containing TMs, such as particulate matter, gaseous pollutants or tobacco smoke, may also pose a risk and cause injury to the lung, liver and kidneys (see Toxicological Profiles from Agency for Toxic Substances and Disease Registry (ATSDR 2021).

**Fig. 1:**
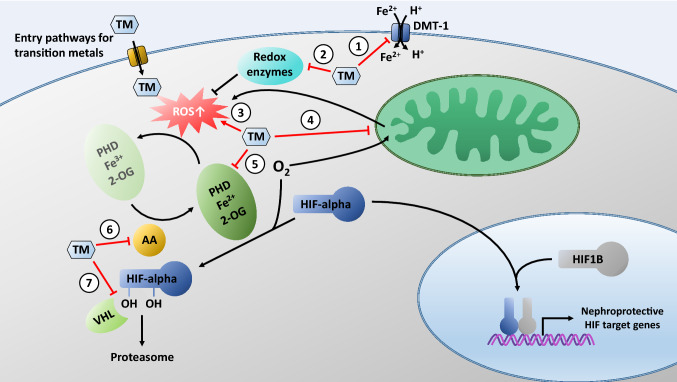
Possible targets of transition metals (TMs) on the HPHE signaling pathway. TMs may enter renal cells via transporters and channels for essential metal ions, such as Fe^2+^. One of these transporters is the divalent metal transporter-1 (DMT-1). TMs may compete with Fe^2+^ and thereby prevent entry of Fe^2+^ via DMT-1, resulting in depletion of intracellular iron needed as a cofactor for PHDs (**1**). TMs contribute to augmented generation of free radicals, either by interfering with reactive oxygen species (ROS) scavenging mechanisms (**2**), by their own redox activity (**3**), or by disrupting the mitochondrial electron transport chain (**4**). Increased ROS inhibit PHDs by oxidizing PHD bound Fe^2+^ to Fe^3+^. TMs may activate HIFs by substituting for Fe^2+^ in PHDs and inactivation of the enzyme (**5**). TMs may deplete intracellular ascorbate (AA) and consequent oxidation of Fe^2+^ to Fe^3+^ in the catalytic center of PHDs (**6**). TMs may stabilize HIF by occupying the VHL-binding domain thereby inhibiting the interaction between VHL protein and hydroxylated HIFs (**7**). For further details, please refer to the text

Quoting the Father of Toxicology, Paracelsus, “*What is there that is not poison? All things are poison, and nothing is without poison. Solely the dose determines that a thing is not a poison*”, this statement is especially relevant for TMs. Up until a certain dose, these metals can be tolerated and are even required by the human body. The kidney is often targeted by increased metal loading due to the plethora of membrane transporters available for crossing the lipid bilayer by molecular mimicry to access the intracellular space where their toxic effects can be unveiled (Bridges and Zalups 2005). Initially, the elevated influx of TMs can be buffered by renal cells through upregulation of chelating proteins, such as metallothionein, or efflux transporters. In addition, general cellular stress responses may be activated to counteract potential damage (Lynes et al. 2007; Weinhouse 2021). However, once the number of binding sites, reservoirs or capacity of storage compartments is exceeded, toxicity occurs. Based on the variable oxidation states and ease of electron donation of TMs, the generation of ROS is a common denominator across TMs and plays a pivotal role in the toxicity execution program (Sabolic 2006) (Fig. 1).

### Free radicals: generation and eradication

The configuration of unpaired electrons in each outer orbital shell of diatomic O_2_ makes it particularly susceptible to free radical formation (Halliwell 1991). Reduction results in an anionic form of O_2_, the superoxide anion O_2_^⋅−^, which is short-lived (Hayyan et al. 2016). Other free radicals generated through reduction of O_2_ include the more reactive hydroxyl radicals (⋅OH) and (hydrogen) peroxide (H_2_(O_2_^⋅−2^)), henceforth referred to as H_2_O_2_ (Munro and Treberg 2017). Though H_2_O_2_ itself is not a free radical, oxidation of iron (Fe^2+^ to Fe^3+^) can generate ⋅OH through Haber–Weiss and Fenton chemistry (Halliwell 1991; Wardman and Candeias 1996). Naturally, iron can be substituted by other TMs. Moreover, superoxide can react with nitric oxide (NO⋅) to form peroxynitrite (ONOO−) and other reactive nitrogen species (RNS). Similar to ROS, RNS are highly reactive and can oxidize thiols and nitrate proteins (Adams et al. 2015).

To limit their damage potential, ROS are either metabolized or neutralized. Superoxide dismustases (SODs) drive the reaction of two superoxide anions with two H^+^ ions to generate the more stable H_2_O_2_ and water (Fukai and Ushio-Fukai 2011; Hayyan et al. 2016). Though H_2_O_2_ can still form ·OH radicals, it can be used as a substrate for several antioxidant enzyme systems, including catalase, glutathione peroxidase, and peroxiredoxins, so that it can be more efficiently removed than superoxide. ROS can be neutralized by glutathione, the most abundant antioxidant, or vitamins. Glutathione is synthesized through a two-enzyme reaction catalyzed by glutamate cysteine ligase and glutathione synthetase. It is a tripeptide nucleophile with thiol groups capable of accepting electrons in its reduced state (GSH), thereby becoming oxidized (GSSG), and rendering ROS to a lesser or non-reactive state (Diaz-Vivancos et al. 2015; Halliwell 1991; Scire et al. 2019; Wardman and Candeias 1996). Both water-soluble (AA, niacin, folic acid) and lipid-soluble (tocopherols) vitamins contribute to the cellular antioxidative capacity. The accumulation and incorporation of tocopherols, eight compounds under the umbrella term of vitamin E (Khadangi and Azzi 2019), into the lipid bilayer (with preference for polyunsaturated fatty acid chains) limits damaging lipid peroxidation.

In addition to modulation and inhibition of antioxidative enzymes and ROS-neutralizing substances, TMs increase ROS levels and oxidative stress by acting directly on mitochondria (Fig. 1). The mitochondrial electron transport chain (mETC) is the largest source of cellular ROS, which are rapidly removed by antioxidants in the mitochondrial matrix. Through the shunting of electrons along four multimeric protein complexes with TM (iron or copper) containing components, electrons can escape and contribute to ROS generation, specifically superoxide anions generated from complexes I and III (Drose and Brandt 2012). Impairment of electron shuttling by damaging complex proteins exposed to elevated levels of TMs culminates in increased ROS production and disruption of physiological ROS signals (e.g. Hosseini et al. 2014; Wang et al. 2004; Xiao et al. 2012).

### ROS: physiological signaling messengers

Whilst unregulated ROS are damaging to cells, driving detrimental effects through unsolicited oxidation of encountered proteins and lipids, it is increasingly apparent that ROS also have physiological functions, such as signal transmission or monitoring of function. How can the cell differentiate between sub-toxic and toxic ROS signals? A key strategy is to compartmentalize ROS, to limit their levels by balancing generation with antioxidative mechanisms, and to control their diffusion capacity. TMs do not only participate in ROS generation as detailed above but can also influence antioxidative capacity, such as by exhausting glutathione supply, inhibiting or damaging antioxidative enzymes, altering protein structure and conformation, and influencing synthesis or regeneration of antioxidants (Dobritzsch et al. 2020; Limon-Pacheco and Gonsebatt 2009). Consequently, physiological ROS can be impacted through disruption, diversion or ablation of the signaling pathway.

Signaling through ROS make take several different routes depending on the TM, its concentration, its route of cellular uptake and intracellular metabolism (Villalpando-Rodriguez and Gibson 2021). Initial stress defense programs could be initiated through low levels of ROS as a sub-toxic load of TM slowly accumulates. This could include the heat shock protein response, prosurvival MAPK signaling, hypoxic response, antioxidative response, unfolded protein response or epigenetic response. Ultimately, toxicity will be elicited through engagement of a cell death program that results in renal injury. Though other forms of cell death may be utilized in specialized cases, such as ferroptosis in the case of iron or autophagic cell death, apoptosis or necrosis are the most likely forms of cell death to be encountered in TM toxicity. The reader is referred to excellent reviews concerning the molecular signaling pathways associated with these cell death forms (Galluzzi et al. 2016; Kalkavan and Green 2018; Lee and Thévenod 2020; Martinou and Youle 2011; Sano and Reed 2013).

## Contribution of mitochondrial ROS to HPHE signaling

Increased production of ROS by cells in a hypoxic environment is counterintuitive and has, therefore, been debated (Clanton 2007). This argument was based on the observation that conditions of hyperoxia lead to elevations in ROS production in many tissues, especially that the amount of superoxide formed from mitochondria is directly proportional to the concentration of O_2_ (reviewed in Jamieson et al. 1986). Nonetheless, as one approaches anoxia, O_2_ availability can become critical for the production of ROS.

Cellular hypoxia is a state that is generally characterized by being in a more cellular reductive state and has been described as a form of “reductive stress” (Dawson et al. 1993). This condition is associated with elevations in reducing equivalents (mostly NADH and FADH_2_) that accumulate in the mitochondria, when there is not sufficient O_2_ is available for reduction by the mETC. The reducing equivalents also make electrons more available for reduction reactions, such as O_2_ to superoxide. Hence the conditions necessary for ROS formation in hypoxia comprise both high reductive capacity (e.g. high NADH/NAD^+^) and sufficient O_2_ available for reaction, whereas in hyperoxia, ROS formation occurs at the expense of low reducing capacity. This bimodal distribution of ROS formation as a function of *P*_O2_ is observed because both hypoxia and hyperoxia support elevations in ROS formation (Clanton 2007).

### Regulation of HPHE signaling by hypoxia-induced mitochondrial ROS

The work of Chandel and Schumacker has provided a link between hypoxia-induced mitochondrial ROS formation and the HPHE signaling axis. Their experimental evidence suggested that the mETC is involved in O_2_ sensing and, therefore, responds to changes in O_2_ levels. In their initial study (Chandel et al. 1998), they used liver Hep3B cells that display transcriptional activation of *EPO*, glycolytic enzymes, and *VEGFA* during hypoxia or in response to cobalt chloride (CoCl_2_) that is a “chemical hypoxia mimic” and are, therefore, commonly used to investigate hypoxia signaling. They tested whether mitochondria act as O_2_ sensors during hypoxia in these cells and whether hypoxia and Co activate transcription by increasing ROS. Wild-type Hep3B cells increased ROS generation during hypoxia (1.5% O_2_) or Co (100 μM) incubation under normoxic conditions for 24 h, whereas Hep3B cells depleted of mitochondrial DNA (ρ^0^ cells), and, therefore, do not respire, failed to increase ROS and to induce mRNA for *EPO*, glycolytic enzymes, or *VEGFA* during hypoxia. In contrast, ρ^0^ cells increased ROS generation in response to Co and retained the ability to induce expression of these genes. Finally, antioxidants abolished transcriptional activation of these genes during hypoxia or Co in wild-type cells and abolished the response to Co in ρ^0^ cells. Thus, the authors concluded that hypoxia activates transcription via a mitochondria-dependent signaling process involving increased ROS, whereas Co activates transcription by stimulating ROS generation via a mitochondria-independent mechanism. In a subsequent study, Chandel et al. (2000) showed that hypoxia increases mitochondrial ROS generation at complex III of the mETC, which causes accumulation of HIF1A protein responsible for initiating expression of a hypoxia-inducible luciferase reporter construct. This response was lost in ρ^0^ cells. Overexpression of catalase abolished hypoxia-induced luciferase expression. Exogenous H_2_O_2_ stabilized HIF1A protein during normoxia and activated luciferase expression in wild-type and ρ^0^ cells. Moreover, isolated mitochondria increased ROS generation during hypoxia. Hence for the first time, these findings revealed that mitochondria-derived ROS are both required and sufficient to initiate HIF1A stabilization during hypoxia.

### Mechanisms of stabilization of HIF-alpha by mitochondrial ROS

These groundbreaking and subsequent studies established that decreasing ROS levels using genetic or pharmacological tools during hypoxia diminishes HIF1A and HIF2A protein levels (Brunelle et al. 2005; Chandel et al. 1998, 2000; Guzy et al. 2005; Lin et al. 2008; Mansfield et al. 2005). However, the questions were still debated whether mitochondria contribute indirectly or directly to HIF-alpha protein stability and which function of the mETC is necessary for HIF-alpha protein stabilization. It had been suggested that under hypoxia, mitochondria, with their high O_2_ consumption, leave the rest of the cell ‘anoxic’. PHDs, would then be deprived of their cofactor, O_2_, leaving them unable to hydroxylate HIF-alpha protein to target it for degradation (Hagen et al. 2003). The second model implicated ROS generation by the mETC as a signaling molecule for HIF-alpha protein stabilization (Bell et al. 2005) and was supported by the above mentioned study (Chandel et al. 2000) as well as by experiments in which cells genetically depleted of cytochrome *c* or the Rieske-Fe-S protein also failed to increase production of ROS during hypoxia (Brunelle et al. 2005; Guzy et al. 2005; Mansfield et al. 2005).

Further studies conclusively identified the complex within the mETC responsible for hypoxic ROS generation and HIF-alpha protein stabilization. Pharmacological evidence indicated that the Qo site of complex III is a likely site of ROS generation during hypoxia (reviewed in Klimova and Chandel 2008). This was validated using cytochrome *b* mutant cybrids, generated by reconstituting 143B ρ^0^ cells with wild-type mitochondrial DNA or that containing a 4-bp deletion in the cytochrome *b* gene found in a patient suffering from parkinsonism (loss of cytochrome *b* renders these cells incapable of O_2_ consumption and unable to generate ROS at the Qi site specifically). However, although respiratory incompetent, these cytochrome *b*-deficient cells were still capable of upregulating hypoxic ROS and stabilizing HIF1A protein (Bell et al. 2007). The mitochondrial antioxidant MITOQ prevented the HIF1A protein stabilization. Furthermore, RNAi to knock down Rieske Fe-S protein to abolish ROS generation at the Qo site in mutant cytochrome *b* cybrids, prevented hypoxic ROS generation and HIF1A protein stabilization, implicating the Qo site of complex III as the key site in hypoxic ROS generation and HIF1A protein stabilization. These cells, however, retained the ability to stabilize HIF1A protein after direct PHD inhibition by DMOG, showing an otherwise intact HIF-signaling pathway. Furthermore, a link between hypoxic ROS generation and hydroxylation of HIF1A protein was established. Neutralizing the ROS with antioxidants allowed HIF1A protein to remain hydroxylated even under hypoxic conditions and, therefore, primed for degradation. On the contrary, increasing ROS levels under normoxia by overexpressing glucose oxidase prevented normoxic HIF1A protein hydroxylation. Altogether, these data demonstrated that the ROS generated by mitochondria under hypoxia prevent hydroxylation of HIF1A protein.

How mitochondrial ROS inactivate PHDs to stabilize the HIF-alpha protein subunit is not understood. One hypothesis is that mitochondrial ROS generated during hypoxia promote the oxidation of cysteine residues within PHD2, resulting in oxidative PHD2 homodimerization and inactivation and leading to HIF1A protein stabilization (Lee et al. 2016). Indeed, PHD2 has several reactive cysteine residues in its C-terminal catalytic domain that may be oxidized by ROS (Briggs et al. 2016; Lee et al. 2016). Interestingly, PHD2 activity requires high intracellular levels of free cysteine, which is regulated by cysteine dioxygenase (Briggs et al. 2016). Free intracellular cysteines may compete with the reactive cysteine residues of PHD2 for ROS-mediated oxidation. Thus, when free intracellular cysteine levels are high, PHD2 cysteine oxidation is prevented. PHD2 is then active, and HIF-alpha protein levels are low. By contrast, limiting the amount of free intracellular cysteine would trigger HIF-alpha protein accumulation. Currently, the significance of these PHD2-reactive cysteines in the stabilization of HIF-alpha under physiological hypoxic conditions remains to be clarified. Metabolites, such as succinate and fumarate, are a second input that inhibits PHD2 activity (reviewed in Lee et al. 2020). To date, no reports have been published that investigated the mechanisms of ROS-induced PHD1/3 inactivation.

In summary, a unifying model to explain HIF-alpha activation is the intrinsic decrease in PHD2 activity owing to declining O_2_ levels coupled with added inputs, such as ROS or metabolites that further diminish PHD2 activity to maximally increase HIF-alpha protein levels.

## Impact of toxic metal ions on the renal HPHE signaling axis

### General considerations

Once toxic metal ions have entered the body they disseminate and accumulate in organs via the blood circulation where they bind to blood cells as well as to various high- and low-molecular weight plasma proteins and peptides. The latter are filtered by the glomerulus and taken up by kidney tubules where they accumulate and damage renal tissue. After acute exposure to high concentrations of metal ions, other organs are damaged as well and kidney injury—likely necrosis—may be indirect and a sequel of pulmonary or cardiovascular problems. Chronic exposure to low concentrations of metals may also lead to renal damage, but the effect is protracted, and ultimately results in chronic kidney disease due to replacement of functional tissue by fibrotic material. The impact on the renal HPHE signaling axis may, therefore, differ between both durations of exposure.

#### TM ion concentration and time dependence of renal HPHE responses

Acute exposure to low TM ion concentrations may trigger activation of (protective) EPO signaling, e.g. by mimicking hypoxia (“chemical hypoxia”), and is either transient or long lasting depending on the exposure time. Protective effects of hypoxia—in addition to Fe, Co, Ni and pharmacological PHD inhibitors—on renal damage induced by various insults have been observed in many studies (Hou et al. 2013; Nath et al. 1992; Nezu et al. 2017; Shimizu et al. 2000; Zager et al. 2016, 2021, 1993). Yet protective effects will only be efficient when interventions occur before the damaging stress, and this strategy has been termed protective “preconditioning” (Wang et al. 2012b). This “(hypoxic) preconditioning” protects organs, including the kidney, against injury, and could be beneficial and improve renal function (reviewed in Bernhardt et al. 2007; Heyman et al. 2011; Shu et al. 2019); see also “Therapy of TM nephrotoxicity, controversies, outlook, and conclusions”). Indeed, the renal HPHE signaling can be stimulated in both acute (e.g. Bernhardt et al. 2006a; Conde et al. 2012; Kudo et al. 2005; Matsumoto et al. 2003; Schley et al. 2011; Schodel et al. 2009; Weidemann et al. 2008) and chronic (e.g. Schley et al. 2019; Tanaka et al. 2005b, c; Theilig et al. 2011) kidney injury, which induces the expression of a variety of tissue protective genes—in particular *EPO* but also *HMOX1*—for adaptation and repair.

Nevertheless, the underlying mechanism is very complex because several hundred genes are targeted by HIFs (Dengler et al. 2014), and time-, isoform- and compartment-specific actions of the HIF pathway seem of utmost importance. But whether activation of the HPHE pathway promotes or antagonizes renal fibrosis elicited by acute kidney injury (AKI), and particularly chronic kidney disease (CKD), is controversial and shows variable outcomes (reviewed in Faivre et al. 2021). Interestingly, a study using a remnant kidney model of CKD in rats has possibly shed some light on these divergent results: Administration of a small molecule inhibitor of PHD dioxygenases (see “Therapy of TM nephrotoxicity, controversies, outlook, and conclusions”) at an early stage accelerated renal fibrosis, whereas at a more advanced stage it decreased renal fibrosis (Yu et al. 2012). Strikingly, the inhibitor given at the early stage activated both HIF1A and HIF2A, whereas given at the later stage it only activated HIF2A with no effect on HIF1A. Consequently, whether HIFs are pro- or anti-fibrotic seems context- and HIF isoform-dependent. The role of the renal HPHE signaling in protection against ischemic kidney injury was investigated in more details using a genetic approach to dissect the contributions of endothelial HIF1A and HIF2A in murine models of hypoxic kidney injury induced by ischemia/reperfusion injury (IRI) or ureteral obstruction (Kapitsinou et al. 2014). In both models, inactivation of endothelial HIF2A, but not endothelial HIF1A, increased expression of renal injury markers and inflammatory cell infiltration in the post-ischemic kidney. Genetic or pharmacologic activation of HIF via HIF prolyl-hydroxylase inhibition protected wild-type animals from ischemic kidney injury and inflammation; however, these protective effects were not observed in HIF prolyl-hydroxylase inhibitor-treated animals lacking endothelial HIF2A. This indicated that endothelial HIF2 mediates protection and recovery from hypoxia-induced renal damage and represents a potential therapeutic target for renoprotection and prevention of fibrosis following acute ischemic injury.

In contrast, high TM ion concentrations may rapidly induce failure of the HPHE system due to cell death and disruption of mitochondrial function (see “Transition metals: environmental presence, exposure, general modes of toxicity in the kidney”; reviewed in Thévenod et al. 2020), thus abolishing physiological ROS signaling and HIF-alpha stabilization (see “Contribution of mitochondrial ROS to HPHE signaling”; reviewed in Lee et al. 2020) (Fig. 1). Chronic exposure to low TM ion concentrations could activate the renal HPHE signaling axis (“preconditioning”; see above) that may delay onset of failure, which occurs after longer periods of exposure and is paralleled by other signs of chronic renal dysfunction. This may occur in organisms, such as humans and experimental animals, but also in cell lines. Hence, seemingly disparate or equivocal observations can be reconciled if concentrations and exposure times are considered. Moreover, the impact of toxic metal ions on renal HPHE signaling may vary depending on whether it occurs in a hypoxic or normoxic environment, or because metal ions may differentially affect O_2_ binding to PHDs.

### Iron (Fe)

#### Exposure and nephrotoxicity

Systemic Fe overload may occur in hereditary hemochromatosis or β-thalassemia, however, the major form of Fe overload is acquired by repeated blood transfusions (Siddique and Kowdley 2012). Systemic Fe overload diseases are associated with chronic damage to a variety of organs, including the heart, liver and endocrine glands. Excess Fe accumulation in these organs is associated with cellular toxicity and death because of its pro-oxidant effects but has also been associated with a number of diseases, and in particular the development of cancer (Toyokuni 2009), wherein excess Fe may cause DNA damage leading to persistent mutations. In addition, Fe is also essential for maintaining the rapid growth rate of cancer cells and may nurture the tumor microenvironment and metastasis. However, Fe can also contribute to cancer defense by inducing toxic ROS and/or initiating specific forms of cell death, including ferroptosis, necroptosis and pyroptosis. Not surprisingly, the carcinogenicity of Fe has been under debate for quite a while (see for instance Huang 2003; Thévenod 2018; Torti et al. 2018; Ying et al. 2021).

The kidney is rarely affected by systemic Fe overload but can be specifically targeted, e.g. in the context of hemoglobin (Hb)-induced AKI subsequent to hemolysis, IRI or due to proteinuria associated with chronic kidney diseases (reviewed in Scindia Ph et al. 2019; Van Avondt et al. 2019; van Swelm et al. 2020). When present in excess and in non-physiologic labile forms, Fe is toxic to the kidneys (as in Hb-associated AKI) and causes renal damage or aggravates AKI elicited by other insults (e.g. Moussavian et al. 2007; van Swelm et al. 2016; Zager and Gamelin 1989; reviewed in van Swelm et al. 2020). Labile (catalytic) Fe is a transitional pool of Fe (Leaf and Swinkels 2016; Slotki and Cabantchik 2015) that is readily available to participate in redox cycling and induces formation of ROS (Halliwell 1991). Through the Fenton reaction (see “Transition metals: environmental presence, exposure, general modes of toxicity in the kidney”) catalytic iron causes oxidative damage to cell membranes, proteins and DNA, which may trigger ER stress (van Raaij et al. 2018; van Swelm et al. 2018) and various forms of regulated cell death, such as ferroptosis (Linkermann et al. 2014) or necroptosis (van Swelm et al. 2018). Yet Fe-induced oxidative damage may be mitigated by nuclear factor erythroid 2-related factor 2 (NRF2)-mediated induction of heme oxygenase-1 (HO-1) (Adedoyin et al. 2018; Alam et al. 2003; Rubio-Navarro et al. 2019; reviewed in Lever et al. 2016; Nath and Agarwal 2020; Tracz et al. 2007). Still the role of Fe in this process appears to be more complex because apart from the obvious injuring effect of Fe on kidney tissue (see above), protective “preconditioning” (reviewed in Bernhardt et al. 2007; Heyman et al. 2011; Shu et al. 2019) by Fe may also occur (see “General considerations” and Hou et al. 2013; Nath et al. 1992; Zager et al. 2016; Zager et al. 2021; Zager et al. 1993).

#### Disruption of HPHE signaling by Fe

However, excess Fe represses the renal HPHE signaling (Oshima et al. 2017; Suzuki et al. 2018). *Epo* gene expression was suppressed in mice following Fe treatment (Oshima et al. 2017). HIF2A (but not HIF1A) was also diminished in the kidney of mice following Fe treatment (2 mg saccharated ferric oxide in a volume of 200 μl per 25 g mouse i.p. for five consecutive days). Moreover, anemia-induced increase in renal EPO and HIF2A expression were inhibited by Fe treatment. Additional cell culture experiments using EPO-producing HepG2 cells showed that Fe stimulation (50–200 µg/ml for 24 h) reduces the expression of the *Epo* gene, as well as HIF2A. Moreover, Fe treatment augmented oxidative stress, and Fe-induced reduction of *Epo* and HIF2A expression was restored by the antioxidant Tempol. HIF2 interaction with the *Epo* promoter was inhibited by Fe treatment and reversed by Tempol. Taken together, these findings suggested that Fe supplementation reduces *Epo* gene expression via an oxidative stress-HIF2A-dependent signaling pathway (Oshima et al. 2017). This was confirmed and extended in a mouse model of EPO-deficient anemia to show that during Fe overload renal interstitial fibroblasts accumulate Fe, and this impairs the hypoxia-driven transcription of the *Epo* gene via renal HIF2 (Suzuki et al. 2018). The authors used “ISAM” (“inherited super anemic mice”) mice, in which both alleles of the *Epo* gene are replaced with the green fluorescent protein (*GFP*) gene, resulting in constitutive activation of the mutant *Epo-GFP* gene in renal EPO-producing cells and hepatocytes due to chronic anemia conditions. By measuring EPO-GFP expression levels in ISAM mice without any specific treatment, the ability of these mice to produce EPO in vivo can be evaluated. Injection of Fe-dextran (10 mg Fe a day for 2 days i.p.) in ISAM mice caused severe Fe deposition in renal interstitial fibroblasts, the site of EPO production. Fe overload induced by either i.p. injection or feeding decreased activity of endogenous *Epo* gene expression by reducing levels of HIF2A. Administration of a Fe-deficient diet to the anemic mice reduced hyperferremia in ISAM mice to normal concentrations and enhanced the ability of renal EPO production. These results demonstrate that Fe overload due to EPO deficiency anemia attenuates endogenous *Epo* gene expression in the kidneys. Thus, iron suppresses EPO production by reducing HIF2A concentration in renal interstitial fibroblasts (Suzuki et al. 2018), indicating that EPO and Fe are in a “conflicted alliance” (Ganz 2018).

In summary, the effects of Fe on the kidney are complex and like a double-edged sword. Fe injures kidney tissues by inducing oxidative stress and various forms of cell death, in particular ferroptosis. At the same time Fe-induced ROS formation activates protective and adaptive signaling pathways, such as NRF2/HO-1 signaling. Whereas hypoxia and Fe may trigger activation of the renal HPHE signaling to protect against acute or chronic kidney injury (“hypoxic preconditioning”; see “General considerations”), excess Fe may also repress HPHE signaling (see Table 2) and thereby prevent induction of tissue protective hypoxia-induced gene products, such as *EPO* and *HMOX1*.

**Table 2:** Effect of TMs on renal toxicity and HPHE signaling (for further details, see “Impact of toxic metal ions on the renal HPHE signaling axis”)

TM	Renal toxicity protocol	Experimental model	Hypoxia/normoxia	Impact on HPHE signaling (↑ ± ↓)	HIFs	PHDs	Target genes investigated	Nephroprotection (↑ ± ↓)	References
Fe	6 days (2 mg Fe_2_O_3_ i.p.1×/day for 5 days)	Mice	Normoxia/anemia	**↓** (fibrosis/myofibroblast transition)	**↓** *Hif1a* / ± HIF1A **↓** *Hif2a* /**↓**HIF2A	n.d	**↓** *Epo*, **↑** *Egln3,* **↑** *Pgk1,* **↑***Vegfa*	**↓**	Oshima et al. (2017)
	4–7 days (10 mg Fe-dextran i.p. 1×/day for 2 days)	ISAM mice (*EPO* gene replaced by *GFP*)	Anemia	**↓** (Fe in interstitial fibroblasts/inflammation)	± *Hif2a* **↓**Nuclear HIF2A	n.d	**↓** *Epo-Gfp*	**↓**	Suzuki et al. (2018)
Co	− 10 to + 3 days (2 mmol/l in drinking water)	45 min ischemia and contralateral nephrectomy in rats	Acute renal ischemia	**↑**	**↑** HIF1A	n.d	**↑** *Hmox1*, **↑** Epo, *↑ Slc2a1, ↑ Vegfa*	**↑**	Matsumoto et al. (2003)
	3–5 weeks (2.7 mg/kg s.c. every 3 days)	Uni-nephrectomized Thy1 nephritis rats	Normoxia	**↑**	**↑** HIF1A	n.d	**↑** *Hmox1*, *↑ Epo*, *↑ Slc2a1, ↑ Vegfa.↑ * SLC2A1, ↑ VEGFA	**↑**	Tanaka et al. (2005b)
	7 days (continuous s.c. infusion of 10 mg/kg/day)	i.m. injection of 80 mg/kg gentamycin 1×/day for 7 days	Normoxia	**↑**	**↑** HIF1A	n.d	**↑** *Vegfa*	**↑**	Ahn et al. (2012)
	30 h (30 mg/kg s.c. 2× every 12 h)	Rats	Normoxia	**↑**	**↑** HIF1A in DT and CD Ø HIF2A	n.d	n.d	n.d	Rosenberger et al. (2002)
	≤ 5 h (≤ 0.1 µmol/ml blood)	Isolated perfused dog kidney	Normoxia/hypoxia	**↑**	n.d	n.d	**↑** EPO (blood)	n.d	Fisher and Langston (1968)
Ni	3–4 weeks (Ni_3_S_2_, 20 mg/rat 1× intrarenally at day 0)	Rats	Normoxia	**↑**	n.d	n.d	**↑** EPO (serum and kidney)	n.d	Hopfer et al. (1984)
Cd	3–12 months (0.5 mg/kg/day i.v. or 2 mg/kg s.c. 1–2×/week)	Rats	Normoxia	**↓** (with inflammation/fibrosis)	n.d	n.d	**↓** EPO/*Epo*	n.d	Hiratsuka et al. (1996); Horiguchi et al. (2000, 2006, 2011)
	14 days (inhalation 2 h/day mist solution 1 mg/ml)	Pregnant rats/fetuses	Normoxia	**↓ (**HIF1 DNA-binding)	**± **HIF1A/*Hif1a*	**± **PHD2/*Egln1*	**↓** *Vegfa*	n.d	Jacobo-Estrada et al. (2018)
Cr	Not studied	Not studied	Not studied	Not studied	Not studied	Not studied	Not studied	Not studied	
Pt	3 days (6 mg/kg cisplatin)	FLAG-tagged luciferase HRE of *Vegf* in Sprague Dawley rats	Normoxia	n.d	↑ *Hif1a*, ↑ *Hif2a*	n.d	↑ *Vegfa*	n.d	Tanaka et al. (2005a)
	3 months (4× weekly 8 mg/kg cisplatin in first 4 weeks)	WT and PT-HIF1a-KO mice	Normoxia	n.d	↑ *Hif1a* in WT	n.d	n.d	↓	Zhao et al. (2021)
	120 h (8 mg/kg cisplatin)	Sprague–Dawley rats	Normoxia or 1% CO	n.d	No change	n.d	n.d	↑ with CO preconditioning	Weidemann et al. (2008)
	24 h 14.7 mg/kg cisplatin	Balb/c mice	Normoxia	n.d			↑ Epo	↑	Eliopoulos et al. (2011)

*i.p./i.v./s.c.* intravenously/intraperitoneally/subcutaneously, *PT/DT/CD* proximal tubules/distal tubules/collecting ducts, *WT/KO* wild-type/knockout, *n.d.* not determined

### Cobalt (Co) and nickel (Ni)

#### Co exposure and nephrotoxicity

Co in hard metal production represents the main source of occupational exposure, e.g. as Co metal dust in the fabrication of tungsten carbide. Other less dominant sources are environmental, dietary, and medical (such as wear and tear of certain metal-on-metal hip prostheses) (reviewed in ATSDR 2004; Leyssens et al. 2017; Simonsen et al. 2012). The evidence for Co nephrotoxicity is weak although it accumulates in the kidney (ATSDR 2004; Simonsen et al. 2012). Rather, Co may be beneficial by attenuating kidney damage induced by various forms of renal insult (see “Iron (Fe)” and below). For instance, in an acute ischemic tubule-interstitial injury model of rats induced by 45-min clamping of renal pedicles with contralateral nephrectomy, elevation of serum creatinine and morphologic injury after the ischemic insult was improved by co-administration of cobalt chloride (2 mM in drinking water from day − 10 to day 3) associated with amelioration of tubulo-interstitial damage and reduction of macrophage infiltration (Matsumoto et al. 2003).

#### Renal HIF-alpha stabilization by Co

In the kidney of rats treated with Co, renal HIF1A protein was upregulated and mRNA or protein levels of several renoprotective genes, such as *Hmox1*, *Epo*, *Slc2a1* and *Vegfa*, were increased before ischemic injury (Matsumoto et al. 2003). In a subsequent study (Tanaka et al. 2005c), the same group applied Co treatment (2.7 mg/kg, subcutaneously, once every 3 days for 3–5 weeks) to uninephrectomized Thy1 nephritis rats, a tubulo-interstitial renal injury model. Although Co did not change glomerular structural damage or urinary protein excretion rate, tubulo-interstitial damage was improved in Co-treated animals and was associated with upregulation of renoprotective HIF-regulated genes (*Hmox1*, *Epo*, S*lc2a1*, and *Vegfa*) as well as increased HIF1A, SLC2A1 and VEGFA proteins. TUNEL staining revealed that the number of apoptotic cells was reduced in the renal cortex by Co administration, suggesting that renoprotection was achieved partly through its antiapoptotic properties. In another study, gentamicin-induced AKI was established in rats by intramuscular injection of 80 mg/kg gentamicin once a day for 7 days (Ahn et al. 2012). Co was continuously infused (10 mg/kg/day for 7 days) into the rats to activate HIF. Co (or DMOG) significantly increased HIF1A expression as well as increased HIF-target gene *Vegfa* and reduced the number of gentamicin-induced apoptotic cells in rat kidneys. HIF activation ameliorated the extent of histologic injury, reduced macrophage infiltration into the tubular interstitium and improved creatinine clearance and proteinuria in gentamicin-induced AKI. An important study was performed by Rosenberger et al. (2002) who investigated the expression of HIF1A and -2A in nephron segments of rats exposed to Co (CoCl_2_ injected subcutaneously twice, at a dose of 30 mg/kg, with a dosing interval of 12 h and animals were euthanized 6 h after the second injection). Proximal tubular cells were negative but marked induction of HIF1A was observed in 70–80% of distal tubular cross-sections, mainly in distal tubules and collecting ducts. In contrast, expression of HIF2A was weak. Unfortunately, no such study was performed with Ni (see below). Interestingly, this nephron distribution of HIF1A matches apical expression of SLC11A2/DMT-1 in the rodent nephron (Ferguson et al. 2001), which transports divalent metal ions with a selectivity of Cd > Fe > Co >> Ni and shows competition of Co with Fe for uptake (Gunshin et al. 1997; Illing et al. 2012). This suggests that the effect of Co on HIF1A is limited by its uptake into nephron cells.

At the cellular level, HK-2 human renal cells were pre-treated for 24 h with Co (150 µM) or DMOG (1 mM) to activate HIF and were then exposed to the nephrotoxic compound gentamicin (3 mM) for another 24 h (Ahn et al. 2012). Co or DMOG significantly increased HIF1A expression in HK-2 cells and inhibited gentamicin-induced ROS formation. HIF1A also protected these cells from gentamycin-induced apoptosis by reducing caspase-3 activity and the amount of cleaved caspase-3, and -9 proteins.

#### Ni exposure and nephrotoxicity

Environmental pollution from Ni may be due to industry (e.g. for alloy production, electroplating, in the production of Ni–Cd batteries and as a catalyst in chemical and food industry), the use of liquid and solid fuels, as well as municipal and industrial waste. Ni-plated water taps may contaminate water and soil; mining and smelting may dump Ni into wastewater; Ni–steel alloy cookware and Ni-pigmented dishes may release Ni into food. Ni contact can cause a variety of side effects on human health, such as allergy, cardiovascular and kidney diseases, lung fibrosis, lung as well as nasal cancer (reviewed in ATSDR 2005; Denkhaus and Salnikow 2002; Genchi et al. 2020). Ni absorbed by humans is excreted by the kidney into the urine. Hence, Ni is not inevitably a cumulative toxin, but larger doses or chronic inhalatory exposure may be toxic, even carcinogenic, and constitute an occupational hazard. When Ni accumulates in the kidney it may or may not induce acute or chronic nephrotoxicity, depending on the dosage and duration of exposure (ATSDR 2005; Das et al. 2008; Denkhaus and Salnikow 2002). Horak and Sunderman exposed rats to inhalation of Ni-carbonyl (0.6 mg/l of air per 15 min), which led to acute toxicity and death of about 20–30% of the animals within 2 h whereas the remaining animals survived for at least 3 days and showed increased proteinuria and aminoaciduria (Horak and Sunderman 1980). Exposure of rats to Ni (44.7–223.5 mg/l via their drinking water for 13 weeks induced a significant decrease in urine volume and an increase in blood urea nitrogen at the highest dose group only, and both immune and pulmonary systems were more sensitive targets than the kidney (Obone et al. 1999). In another chronic nephrotoxicity study, rats were given 100 mg/l of Ni (as Ni–sulfate) in drinking water for 6 months which resulted in increased urinary excretion of albumin (but not *N*-acetyl-β-d-glucosaminidase (NAG) or β2-microglobulin), suggesting glomerular rather than tubular damage (Vyskocil et al. 1994).

#### Activation of renal HPHE signaling by Co and Ni through chemical hypoxia

When hypoxia causes increased ROS formation (Lee et al. 2020), this triggers HO-1 expression (see “Iron (Fe)”; also reviewed in Agarwal and Nick 2000; Gozzelino et al. 2010). Co and Ni rapidly induce renal microsomal HO-1 under normoxic conditions, similarly as other TM ions, such as Cd or Pt (see “Platinum (Pt)”; reviewed in Agarwal and Nick 2000; Sunderman 1987). Because HO-1 induction by metals was generally suppressed by treatments with SH compounds (for example, cysteine and glutathione) and enhanced by agents that deplete tissue SH levels (for example, diethyl maleate), Sunderman concluded that the induction mechanism may involve binding of metal ions to SH-containing regulatory molecules. However, oxidative stress induced by metal ions, which affects the redox signature of kidney tissue (Stohs and Bagchi 1995) and results in cytoprotective induction of HO-1, is the other side of the coin (reviewed in Agarwal and Nick 2000; Gozzelino et al. 2010), where increased HO-1 may be mediated by HIF induction as well (Lee et al. 1997). Cell culture experiments support oxidative stress induced by Co and Ni. Ni (1–500 µM for 12–72 h) increased the formation of ROS, lipid peroxidation, apoptosis and DNA damage in normal rat kidney (NRK) cells (Chen et al. 2010). Along the same lines, exposure of HK-2 cells to Ni (160–480 µM for 12–72 h) increased ROS, apoptosis, and DNA damage, which were prevented by pretreatment with *N*-acetylcysteine (Wang et al. 2012a).

Co and Ni caused EPO production in dog and rat kidneys after acute (4 h for Co) or chronic exposure (3 weeks for Ni), respectively (Fisher and Langston 1968; Hopfer et al. 1984). At the cellular level, most mechanistic studies were performed on the human hepatoma cell line Hep3B, which regulates its production of EPO in a physiologic manner. Other studies mainly used respiratory human non-cancerous or cancerous cell lines because of the entry route of Co and Ni by inhalation (ATSDR 2004, 2005). No studies have been performed on kidney cell lines so far. Therefore, it is difficult to estimate whether the observations made in liver and respiratory cells are valid for the kidney. Historically, evidence for a mechanism common to hypoxia, Co, and Ni was obtained by demonstrating increased EPO production in Hep3B cells (Goldberg et al. 1988). Inhibition of EPO production at low partial pressures of O_2_ by carbon monoxide indicated that a heme protein is integrally involved in the O_2_-sensing mechanism. Moreover, when heme synthesis was blocked, hypoxia-, Co- and Ni-induced EPO production were all inhibited. The authors concluded that EPO is induced by hypoxia or Fe depletion and that its induction by the TMs Co or Ni results from substitution of the Fe atom in an “O_2_ sensor” (Goldberg et al. 1988). The most likely explanation at that time (which turned out to be inaccurate) was that the O_2_ sensor is a heme protein in which Co and Ni can substitute for Fe in the porphyrin ring, whereby Fe-protoporphyrin would bind O_2_ with high affinity, Co-protoporphyrin with low affinity and Ni-protoporphyrin not at all (reviewed in Huang et al. 1997).* EPO* transcription is now known to be under the control of HIF proteins (Semenza and Wang 1992). In response to 75 µM CoCl_2_, *HIF1A* and *HIF1B* mRNAs of Hep3B cells peaked at 4 h, declined at 8 h, and increased again at 16 h (Wang et al. 1995). Exposure of human osteosarcoma (HOS) cells and a Ni-transformed derivative, SA-8, as well as MCF-7 and A549 human cancer cells to Co (0.2 mM) or Ni (1 mM) for 6–24 h has also been shown to induce HIF1A (Salnikow et al. 1999).

Maxwell and Salnikow (2004) have reviewed various mechanisms that could underlie “chemical hypoxia” associated induction of HIF-alpha elicited by Co and Ni. One means by which Co and Ni may activate HIF-alphas was that they substitute for Fe in regulatory HIF dioxygenases, the PHDs (see Epstein et al. 2001; Ivan et al. 2001; Jaakkola et al. 2001 and “HIFs/PHDs: isoforms, regulation, tissue specific expression, mechanisms in the kidney”), and this substitution inactivates the enzymes (see above), as already demonstrated with phthalate dioxygenase (see above and Batie et al. 1987). Another mechanism was proposed following in vitro experiments demonstrating that Co binds with high affinity to HIF2A via sites within the ODD domain in an O_2_-dependent manner and that the Co binding site overlaps with the VHL-binding site of HIF. Hence, Co and other TMs may disrupt the interaction between VHL protein and HIFs by directly binding to hydroxylated HIFs in vivo (Yuan et al. 2001). An alternative explanation to direct substitution for Fe in the regulatory dioxygenases was that Ni or (less strongly) Co could bind more tightly than Fe^2+^ to the membrane transporter SLC11A2/DMT-1 (Gunshin et al. 1997; Illing et al. 2012) and suppress delivery of ferrous iron into cells. Another option was that Co and Ni produce increased ROS levels in cells (see “Transition metals: environmental presence, exposure, general modes of toxicity in the kidney” and Fig. 1), which may stabilize HIF-alpha because (mitochondrial) ROS play an important role in HIF-alpha stabilization (see “Contribution of mitochondrial ROS to HPHE signaling”; reviewed in Lee et al. 2020). Oxidative stress would either inactivate the PHDs directly or indirectly by AA depletion, which is necessary for optimal function of the enzymes. Alternatively, direct interaction of metals with AA could prevent entry of AA into cells resulting in depletion of intracellular AA (reviewed in Maxwell and Salnikow 2004).

#### Mechanisms of PHD inhibition by Co and Ni

Most publications investigating the role of Co and Ni in HPHE signaling were performed in human airway normal or carcinoma cell lines, although the conclusions drawn from these studies may not be applicable to the kidney, but the main results are summarized as follows. Undoubtedly, Co and Ni are good inducers of HIF-alpha, and ROS are produced during the exposure of cells to these TMs. However, Salnikow and colleagues concluded that the formation of ROS is not involved in HIF stabilization or the activation of HIF1-dependent genes (Andrew et al. 2001; Salnikow et al. 2000a, b) (in contrast, see “Contribution of mitochondrial ROS to HPHE signaling” and Chandel et al. 1998 for different results obtained in Hep3B cells). One weakness of all these studies is that ROS formation was assayed using dichlorodihydrofluorescein diacetate, which cannot be reliably used to measure intracellular H_2_O_2_ and other ROS (see Dikalov and Harrison 2014). Hence, Salnikow et al*.* proposed that in addition to low oxygenation, Co, Ni (and possibly other TMs) deplete intracellular AA by excessive oxidation or insufficient supply of AA (due to inhibition of the AA transporter SVCT2) causing inhibition of PHDs, which are dependent on AA (Kaczmarek et al. 2007; Karaczyn et al. 2006; Salnikow et al. 2004). The role of AA is to reduce PHD enzyme-bound Fe, which is important for maintaining hydroxylase activity (Kaczmarek et al. 2009). The latter study indicated that the energy required for Fe substitution by Ni or Co in the enzyme is too high to be achieved in a biological system, thus contradicting the model favoring Fe replacement. Additionally, computer modeling identified a tridentate coordination of AA with the enzyme-bound Fe, which would explain the specific demand for AA as the Fe reductant. Thus, these data supported the hypothesis of Fe oxidation in the hydroxylases following exposure to TM ions (Kaczmarek et al. 2009). In contrast, using airway epithelial cell models as well the group of Costa and colleagues provided evidence that Ni and Co replace Fe in the hydroxylases and interfere with Fe uptake (Chen et al. 2005; Davidson et al. 2005, 2006; Li et al. 2006). Costa and coworkers (Davidson et al. 2006) demonstrated that Ni stabilizes HIF1A and decreases VHL binding to the ODD domain of HIF1A. Furthermore, Ni inhibited cellular PHDs as well as purified PHD2 in vitro through direct interference with the enzyme. Through theoretical calculations, the authors demonstrated that Ni may be able to replace the Fe in the active site of this enzyme. Hence, in contrast to the work of Salnikow et al*.* (Kaczmarek et al. 2009, 2007; Karaczyn et al. 2006; Salnikow et al. 2004) these authors concluded that Ni can interfere with PHDs directly and does not inhibit the enzyme by depleting cellular factors, such as Fe or AA (Davidson et al. 2006).

In summary, chronic Ni accumulation in the kidney may disrupt renal HPHE signaling axis by causing cell death and fibrosis, whereas Co may be beneficial by attenuating kidney damage induced by various forms of renal insults, which is consistent with the hypothesis of protective hypoxic "preconditioning". Whereas various animal studies have investigated Co effects on HPHE signaling, no such studies have been published for Ni (see Table 2 for a summary). At the cellular level, no studies have been performed on renal cell lines. Strikingly, there are few experimental data showing Co/Ni dependent stabilization of HIF2A. The underlying mechanism of HIF-alpha stabilization remains a matter of debate and may involve iron substitution of PHDs, which inactivates the enzymes, disruption of the interaction between VHL protein and HIFs by directly binding to the ODD domain of HIFs, or ROS formation with consequent AA depletion and subsequent iron oxidation (see Fig. 1). Considering the current literature, it is difficult to explain why Ni should be more nephrotoxic than Co only on the base of their interaction with the (nephroprotective) renal HPHE signaling axis. Hence other factors will have to be considered, including the distribution of putative transporters for Co and Ni in different nephron segments, the affinities of Co and Ni to those transporters, their role in iron depletion or displacement, their impact on the antioxidative status of tubule cells, etc., and all these factors may account for differential Co and Ni nephrotoxicities.

### Cadmium (Cd)

#### Exposure and nephrotoxicity

Contamination of the environment by Cd may occur through anthropogenic and natural sources (WHO 1992, 2010). Food and drinking water are the main routes of exposure to Cd for the nonsmoking general population. Although the efficiency of Cd absorption through inhalation (25–50%) is much higher than that through ingestion (1–10%), concerns about airborne exposure is limited to special populations, including smokers, people living near smelters, and metal-processing workers. In contrast, dietary Cd intake is an important public health issue for the general world population despite the lower bioavailability of Cd through the gastrointestinal tract. The real challenge setting seems to be the chronic (i.e., over decades or even throughout life) low (i.e., in concentrations barely exceeding the “natural” environmental Cd concentrations) Cd exposure (CLCE) from dietary sources and cigarette smoking. Modern agriculture globally uses Cd-containing phosphate fertilizers to increase the efficiency of harvests. Plants, including tobacco, accumulate Cd, which is passed on to animals and man in the food chain (reviewed in Moulis and Thévenod 2010; Thévenod and Lee 2013). Cd in tobacco smoke takes a share in the development of smoking-associated chronic ailments, such as cardiovascular diseases or diabetes mellitus. Moreover, Cd is a class I carcinogen because it interacts indirectly with DNA consequent to elevated ROS levels, interferes with major DNA repair systems, as well as inactivates tumor suppressor functions by targeting proteins with Zn-binding structures. This may cause genomic instability and promote tumor initiation and progression. Cd is stored in various organs, and particularly in the kidney, with a half-life of several decades. Hence, CLCE damages multiple organs in humans and other mammalian organisms by causing nephrotoxicity, osteoporosis, neurotoxicity, genotoxicity, teratogenicity, or endocrine and reproductive defects (Thévenod 2003, 2009; Thévenod and Lee 2013).

#### Disruption of renal EPO production by Cd

An early study investigated nephrotoxicity and anemia in rats exposed to Cd (0.05 or 0.5 mg/kg b.w.) by intravenous application for 50 weeks (Hiratsuka et al. 1996). In the high Cd group, renal tubular disorders became marked at 16 weeks and cortical fibrosis with glomerular dysfunction appeared at 50 weeks. Anemia occurred at 12 weeks and worsened over time. Early on, EPO levels increased as the hemoglobin level decreased, indicating intact renal HPHE signaling axis. In contrast, EPO levels were not elevated at 50 weeks despite marked iron deficiency anemia, indicating that chronic renal damage and fibrosis disrupted the renal HPHE signaling axis (Hiratsuka et al. 1996). Concomitantly, another group confirmed these data (Horiguchi et al. 1996). Rats were exposed to 2.0 mg Cd/kg b.w. by subcutaneous injection once a week for 6 or 9 months and showed anemia with low levels of plasma EPO over time along with hypoinduction of *Epo* mRNA in the kidneys, which were accompanied by biochemical and histological renal tubular damage. The results indicated that chronic Cd-intoxication causes anemia by disturbing the EPO production capacity of renal cells (Horiguchi et al. 1996). In a subsequent study, the same group investigated the local relationship between hypoxia-induced EPO production and renal tubular injury in control rats and rats injected with Cd at 2 mg/kg twice a week for 8 months (Horiguchi et al. 2006). Anemia due to insufficient production of EPO was observed in chronic Cd-intoxication of rats. In situ hybridization detected *Epo* mRNA expression in proximal tubule (PT) cells of hypoxic control rats, but Cd-intoxicated rats showed atrophy of EPO expressing PT and tissue fibrosis (similar effects were observed a single dose of cisplatin at 8 mg/kg, which targets PT). The authors concluded that Cd (and cisplatin) induces anemia through direct damage of PT cells that mainly contribute to renal EPO production (peritubular fibroblasts-like interstitial cells did not express *Epo* mRNA in this study; however, see Nagai et al. 2014 for a reevaluation of EPO production by the nephron). Using the same Cd-intoxication protocol, these authors also investigated the role of Cd-induced hemolysis on renal damage in rats (Horiguchi et al. 2011). Renal iron overload was observed after 3 months of Cd application and was accompanied by increased urinary levels of NAG, glucose, transferrin, and hemoglobin that are all indicative of PT damage. Furthermore, increased renal expression of *Il6* indicated increased inflammation of damaged renal tissue. The authors concluded that in addition to direct damaging effects of Cd on PT, Cd-induced hemolysis leads to Fe accumulation in the kidneys with resulting oxidative stress and cell death (see also “Iron (Fe)”), which aggravates renal damage and decreases EPO production, e.g. by destruction of EPO-producing cells (Horiguchi et al. 2011).

#### Roles of Cd in HPHE signaling

Cell culture studies have been performed in an attempt to determine mechanistically the interference of Cd with HPHE signaling (Chun et al. 2000; Gao et al. 2013; Horiguchi et al. 2000; Jing et al. 2012; Li et al. 2006; Obara et al. 2003), yet no study with renal cell lines has been published so far, which limits the conclusions drawn from these studies for the kidney (see also “Cobalt (Co) and nickel (Ni)”). Moreover, it is difficult to estimate whether the acute effects of exposure to submicromolar or low micromolar concentrations of Cd for up to 24 h in cell lines can be extrapolated to the more chronic effects observed after systemic application of Cd for weeks or months in vivo. Most importantly, only recently has an in vivo study determined the impact of chronic Cd exposure on several components of the renal HPHE signaling axis (Jacobo-Estrada et al. 2018) and was able to provide information at the same biochemical level as the cell culture studies. In most studies with Cd cited above, hypoxia was used a stimulus to activate HPHE signaling (Chun et al. 2000; Horiguchi et al. 2000; Obara et al. 2003), but some investigators triggered “chemical hypoxia” with Co (Gao et al. 2013; Horiguchi et al. 2000) or determined the effect of Cd under normoxic conditions (Jing et al. 2012; Li et al. 2006). Several cell culture studies with the human hepatoma cell line, Hep3B, found that Cd at concentrations and exposure times that do not affect cell viability (0.5–50 µM for 16–24 h) inhibits HIF1A DNA-binding activity (Chun et al. 2000; Horiguchi et al. 2000; Obara et al. 2003). This observation is relevant for the kidney because inhibition of HIF1A DNA-binding was recently confirmed in vivo in embryonic kidneys after exposure of pregnant rats to Cd (by inhalation for 2 h/day to a mist of a solution of 1 mg CdCl_2_/ml from gestational day 8–20) and removal of embryonic kidneys at day 21 (Jacobo-Estrada et al. 2018). Accordingly, expression of hypoxia-inducible target genes, such as *Epo* or *Vegfa*, or hypoxia-induced luciferase reporter gene activity were reduced by Cd (Chun et al. 2000; Horiguchi et al. 2000; Jacobo-Estrada et al. 2018; Obara et al. 2003). However, the effect of Cd on *HIF1A* mRNA and HIF1A protein is less consistent in the literature. Chun et al*.* (2000) observed no change of *HIF1A* mRNA but decreased HIF1A protein, which they attributed to Cd stimulation of proteasomal activity, whereas Jacobo-Estrada et al*.* (2018) did not observe any effect of Cd on mRNA or protein levels of HIF1A and PHD2. Interestingly, using a recombinant catalytic domain of human PHD2 and non-denaturing ionization electrospray mass spectrometry, Mecinovic et al*.* have demonstrated Cd binding to the apo-enzyme at the catalytic site plus at a novel binding site (as well as with Zn, Cu and Co, but not Fe or Ni) (Mecinovic et al. 2008). Cd could partially displace Fe from the PHD2 active site, suggesting Cd inhibition of PHD2. These in vitro data are in contrast to the above in vivo and cellular studies where Cd showed no increased HIF-alpha stabilization as well as to studies proposing AA depletion as the sole mechanism underlying PHD2 inhibition and excluding Fe displacement by other TMs (see “Cobalt (Co) and nickel (Ni)”; reviewed in Maxwell and Salnikow 2004).

Because Cd intake by organisms occurs through inhalation in addition to the oral route, Cd effects on HPHE signaling were also studied in airway epithelial cell lines. However, the results were variable, possibly because of differences in Cd concentrations, exposure times and cell lines used. Exposure of chemically hypoxic (Co treatment) rat lung fibroblasts (RFL6) to Cd resulted in inhibition of HIF1A DNA binding to the hypoxia-inducible gene lysyl oxidase *Lox* and reduced *Hif1a* mRNA expression (Gao et al. 2013). Other studies showed either no effect of Cd on HIF1A expression in normoxic cells (Li et al. 2006) or increased HIF1A and *VEGFA* expression under normoxia (Jing et al. 2012).

In summary, in renal tissue Cd may disrupt the renal HPHE signaling axis by interfering with HIF1A binding to DNA of hypoxia-inducible target genes and/or decrease *Hif1a* mRNA and protein expression, resulting in decreased renal EPO production and ensuing renal anemia, which may aggravate Cd-induced renal injury by disrupting nephroprotective HPHE signaling. To the best of our knowledge, no study has addressed the effect of Cd on (renal) HIF2A expression. Table 2 summarizes data from animal studies.

### Chromium (Cr)

#### Exposure and nephrotoxicity

Hexavalent Cr (Cr^6+^) is often found in occupational settings as a by-product of various industrial processes, such as leather working, smelting, welding, and metal plating (ATSDR 2012). Cr^6+^ is also found in automobile exhaust and in tobacco products, such as traditional and electronic cigarettes and hookahs, (Williams et al. 2017) and is, therefore, a human respiratory carcinogen, and produces a variety of toxic effects through inhalation (ATSDR 2012). Systemic toxicity attributable to Cr^6+^ has been documented in the respiratory and pulmonary system, gastrointestinal tract, dermis, and renal system (ATSDR 2012). Contact dermatitis is frequently documented following exposure to chromate and dichromate (Lejding et al. 2018). Exposure to Cr^6+^ can also cause acute tubular necrosis, which is localized to the proximal convoluted tubules and may result in rapid onset of renal failure (Wedeen and Qian 1991). In addition, multiple mechanisms of Cr^6+^ carcinogenesis have been proposed involving oxidative stress, DNA damage and genomic instability, and epigenetic modulation (reviewed in Chen et al. 2019b). Cr^6+^ compounds, such as chromate and dichromate, are strong oxidizing agents at low or neutral pH. The product of Cr^6+^ reduction, Cr^3+^, is rather non-toxic and has even been considered an essential nutrient in humans for insulin, sugar, and lipid metabolism.

The mechanisms of Cr-associated nephrotoxicity are only partly elucidated. In rats, Cr selectively accumulates in the renal cortex at 6–20 times the level present in red blood cells or the liver (Weber 1983). Cr may induce nephrotoxicity in humans. However, tubular damage following occupational exposure is mostly due to acute absorption and transient in nature (Franchini and Mutti 1988; Sharma et al. 1978). Experimental studies in rats support acute Cr nephrotoxicity caused by oxidative stress (Gumbleton and Nicholls 1988; Ngaha 1981; Patlolla et al. 2009; Pedraza-Chaverri et al. 2005; Seiken et al. 1994; Zhou et al. 2008). The involvement of different nephron segments in Cr-induced oxidative stress and toxicity was assessed in AKI induced by K_2_Cr_2_O_7_ (Arreola-Mendoza et al. 2006). Rats received K_2_Cr_2_O_7_ (a single dose of 15 mg/kg, s.c.). Altered PT function, decreased glomerular filtration, and distal segment dysfunction were accompanied by oxidative damage 48 h after exposure to Cr. In α-tocopherol-treated animals (125 mg/kg by gavage 5 days before and during Cr exposure) proximal reabsorptive and secretory functions were preserved, implying that oxidative damage contributes to Cr toxicity. In contrast, glomerular or distal dysfunction were not prevented by α-tocopherol, suggesting nephron segment specificity of Cr-induced oxidative stress.

Chronic Cr nephrotoxicity has also been described in humans and experimental animals. Nuyts et al. (1995) observed that occupational chromium exposure increases the risk of chronic renal failure by about threefold in industrial areas, which was confirmed in subsequent studies (Tsai et al. 2017; Wang et al. 2011). Animal experiments are consistent with these observations and indicate chronic Cr nephrotoxicity induced by oxidative stress (Soudani et al. 2010). In another study, K_2_Cr_2_O_7_ administrated to female rats during late pregnancy and early postnatal periods provoked kidney damage mediated by oxidative stress in dams and their offspring (Soudani et al. 2011). Malondialdehyde (an indicator of lipid peroxidation), GSH and NO levels increased in kidneys of Cr-treated mothers and their suckling pups. Activities of SOD, CAT and glutathione peroxidase were increased in dams and decreased in their pups. Significant decrease in creatinine clearance was also found in treated mothers and in their progeny.

At the cellular level, a few studies have investigated mechanisms of acute Cr toxicity in cultured renal cells. Dartsch et al. (1998) compared opossum kidney and HepG2 liver cells because acute Cr nephrotoxicity is known to be more prominent than hepatotoxicity in vivo. Cr^6+^ (0.01 µM to 1 mM for 24 h), but not Cr^3+^, had a dose-dependent cytotoxic effect with loss of cell viability *(EC*_50_ ~ 5 µM for kidney and ~ 50 µM for liver epithelial cells). Chloride channel blockers did not inhibit cell damage, suggesting that the uptake of Cr^6+^ did not occur through anion transporters. In another study, the contribution of oxidative damage was investigated in HK-2 cells incubated with 10 μM K_2_Cr_2_O_7_ for 24 h (Lin et al. 2018). Supplementation with AA (30 μg/ml) inhibited damage to HK-2 cells, if incubated within 1–8 h of Cr toxicity by preventing ROS generation, apoptosis, and autophagy, as well as Cr entry into cells, possibly by reduction of Cr^6+^ to Cr^3+^.

#### Mechanisms of Cr^6+^-induced HIF-alpha stabilization

Salnikow and colleagues tested their hypothesis that TM ions can induce HIF-alpha by depleting intracellular AA via oxidation and by inhibiting AA uptake by cells (Karaczyn et al. 2006; Salnikow et al. 2004). Cr^6+ ^is known to oxidize AA directly (Zhitkovich 2005) and is a principal AA oxidant in rat liver and kidneys (Standeven and Wetterhahn 1991). Salnikow and coworkers (Kaczmarek et al. 2007) compared the accumulation of HIF1A protein in human lung epithelial cells (1HAEo- and A549) following Ni (500 µM) or Cr^6+^ (5 µM) exposure. The experiments showed that there is a difference in the time course of this accumulation. Thus, HIF1A protein was induced only by Cr^6+^ at 1–8 h and disappeared after the latter was reduced to Cr^3+^. This correlated with cell-free experiments showing a rapid phase of AA oxidation by Cr^6+^, which ends after the completion of Cr^6+^ reduction to Cr^3+^. In contrast, extended stabilization of HIF1A was observed following acute exposure to Ni for up to 24 h. Ni was found to be a catalyst, which facilitates continuous oxidation of AA by ambient O_2_. A HIF1A-dependent reporter assay revealed that 20–24 h was required to fully develop HIF1 transcriptional response and the acute exposure to Ni, but not Cr, met this requirement. However, repeated (chronic) exposure to Cr^6+^ also led to extended stabilization of HIF1A. Thus, these data emphasized the important role of AA in regulation of HIF1 transcriptional activity in metal-exposed human lung cells. In a very recent study, another aspect of the regulation of HPHE signaling was investigated in HepG2 cells exposed to Cr (Nishimura et al. 2021). At variance with the report by Kaczmarek et al. (2007), the authors reported that Cr^3+^ (100 µM for 24 h) increases HIF1A protein, *EPO* mRNA expression and EPO protein levels in HepG2 cells. The effect of Cr on EPO production was abolished by co-incubation with the inhibitor of proliferator-activated receptor γ (PPARγ), SR-202 (100 µM), suggesting a different mode of action of Cr^3+^ on HIF1A protein levels that was mediated by PPARγ induction of HIF1A, and which is supported by other studies (Tsave et al. 2016; Urakami-Takebayashi et al. 2018; Zhou et al. 2009).

#### Epigenetic mechanisms in HPHE signaling affected by Cr^6+^

Hypoxia-induced HIF1A stabilization is known to regulate epigenetic mechanisms, e.g. by promoting histone demethylation (Beyer et al. 2008; Krieg et al. 2010; Pollard et al. 2008; Wellmann et al. 2008; Xia et al. 2009), which may act as signal amplifiers to facilitate hypoxic gene expression and ultimately enhance tumor growth (reviewed in Hancock et al. 2015; Watson et al. 2010). Costa and coworkers (reviewed in Chervona et al. 2012) proposed that Cr (as well as Ni) may induce post-translational histone modifications and affect the enzymes that modulate them, i.e., members of the Fe- and 2-oxoglutarate-dependent dioxygenase family, including PHD2, histone demethylases JHDM2A/JMJD1A, and DNA repair enzymes ABH3 and ABH2, as well as histone methyltransferases, G9a. Hence, given the effects that these metals may exert on the epigenome, their involvement in the dynamics of histone modifying enzymes could also account for their respective toxicities and carcinogenicities.

In summary, there is ample evidence that Cr^6+^ causes acute and chronic nephrotoxicity. Strikingly, no studies are available studying the impact of Cr^6+^ on HPHE signaling in the kidney or kidney cell lines. As a “chemical hypoxia mimetic” Cr^6+^ is a HIF1A stabilizer, but the underlying mechanism is not clear because only few studies have investigated this process, although it may not displace Fe from PHDs. The role of Cr^6+^ on other aspects of HPHE signaling (e.g. on HIF2A) and on nephroprotection remains to be investigated.

### Platinum (Pt)

#### Exposure and nephrotoxicity

Platinum is mainly used in the automobile industry for autocatalysts, sensors and spark plugs. Other uses in industrial applications, such as in electronics, and jewelry make up the worldwide demand for this metal (Rauch and Morrison 2008). Interactions of platinum compounds with biological systems have been well reported in the literature. It was first observed that bacterial, viral and fungal pathogens could be rendered nonviable by platinum metals (Cochran and Maassab 1970; LeRoy 1975; Rosenberg et al. 1967; Shulman and Dwyer 1964). Based on these observations, platinum compounds were tested in leukemia and sarcoma cancers, which were implanted into mice. Of the four metal-amine complexes reported in the study, *cis*-Pt(II)(NH_3_)_2_Cl_2_, commonly known as cisplatin or Peyrone’s salt, was the most effective in reducing sarcoma tumor mass (by > 95%) or prolonging mean survival time (by > 80%) in the leukemic mouse model (Rosenberg et al. 1969). Moreover, the mice remained cancer-free for six months post-treatment. By 1972, phase I clinical trials with cisplatin were completed and in 1978, it was approved by the FDA for treatment of testicular, ovarian and bladder cancer. Cisplatin therapy is tied with toxicities predominantly in the kidney, intestinal tract and ear (Barabas et al. 2008; Karasawa and Steyger 2015; Qi et al. 2019), due to uptake of the drug by membrane transporters (Ciarimboli 2014), in particular organic cation transporters (Ciarimboli et al. 2005; Zhang et al. 2006), which are prevalent in these tissues (Lee et al. 2009). Nephrotoxicity seems to be the limiting factor in therapeutic dosing of cisplatin. Despite this and development of drug resistance, cisplatin is widely used to treat a number of solid tumors. A number of less toxic analogs have since been developed, including carboplatin and oxaliplatin (Sharma et al. 2022).

Cisplatin’s square-planar configuration predominantly undergoes nucleophilic substitution and, therefore, permitting its activation by displacement of the chloride ions by water molecules. Hydrolyzation of cisplatin results in a potent electrophile, which reacts with sulfhydryl groups on proteins and nitrogen donor atoms on nucleic acids (Dasari and Tchounwou 2014; Sharma et al. 2022). Hence, cisplatin’s antitumor action is not dependent on redox reactions of platinum itself. Rather, it attacks several intracellular targets, such as deoxyribonuclease I (Basnakian et al. 2005), involved in DNA and protein syntheses as well as intercalating into DNA, reducing Na^+^/K^+^-ATPase activity and total GSH levels (Courjault et al. 1993) to render its cytostatic actions. Of note, these aforementioned mechanisms appear to be specific to the antitumor action of cisplatin whereas the toxic effects are primarily consequent of oxidative stress due to mitochondrial dysfunction.

#### Role of cisplatin and HPHE signaling in renal injury

Hypoxia and HIFs have been implicated in both cisplatin-induced injury and advancement of drug resistance in the kidney (reviewed in Li et al. 2021). Following cellular uptake, cisplatin elicits an inflammatory response as well as mitochondrial dysfunction and decreasing antioxidative capacity, which culminates in increased levels of ROS and renal cell injury (reviewed in Holditch et al. 2019; McSweeney et al. 2021; Volarevic et al. 2019). The exact role of hypoxia and HIFs in renal damage elicited by cisplatin is hampered by conflicting data. In immortalized PTCs, hypoxia protected against cisplatin-induced apoptosis by attenuating Bax accumulation and cytochrome *c* release yet was independent of the activated HIF1A (Wang et al. 2006). Rather, the authors postulated a role for p53 activation by cisplatin that is suppressed by hypoxia, and inhibition of the mETC (Wang et al. 2006). Conversely, cisplatin-induced apoptosis was augmented by hypoxia in a cell line resembling principal cells of the renal collecting duct (Schwerdt et al. 2005). Cisplatin concentrations up to 30 µM did not induce proapoptotic caspase-3 activity whereas co-incubation with mETC inhibitors activated caspase-3 by up to 20-fold over controls and initiated DNA laddering. Inhibition of the mETC (to mimic hypoxic conditions), but not cisplatin per se, increased lactic acid thereby reducing intracellular pH and potentiated caspase-3 activation. Furthermore, caspase-3 activity was elevated by > twofold in cisplatin-treated cells under severe hypoxic conditions. These conflicting studies could be explained by the cell models (proximal tubule versus collecting duct) used wherein the HIF turnover is regulated differently based on the O_2_ tension. Due to physiological low O_2_ tension in the renal medulla, wherein collecting ducts are located, higher expression levels of PHD2 and PHD3 were detected (Schodel et al. 2009), presumably to maintain low HIF levels. Cisplatin treatment in Sprague–Dawley rats depreciated total PHD2 and PHD3 expression in parallel with increased blood creatinine, though in the absence of HIF induction, indicating HIFs are not essential to renal injury progression, which has been confirmed in resistant cell clones following repeated episodes of hypoxia (Brooks et al. 2007). Thus, whether hypoxia protects against or exacerbates cisplatin nephrotoxicity depends on the origin of the renal cells and does not seem to involve HPHE signaling.

The activation of HIFs following cisplatin is also unresolved. Tanaka et al. (2005a) observed increased HIF expression in the outer medulla three days after cisplatin administration to rats and Zhao et al. (2021) detected HIF1A in mouse renal tubules and primary and immortalized PTCs post-cisplatin treatment whereas Weidemann et al. (2008) did not find increased HIF1A in immortalized PTCs or rat renal tubules after exposure to cisplatin. Only hypoxic preconditioning with 0.1% carbon monoxide (without cisplatin) led to marked increase in HIF1A, in particular in the outer medulla (Weidemann et al. 2008), coincidently as observed by Tanaka et al. (2005a). In both studies, elevated HIF1A protected against cisplatin-induced tubular cell apoptosis and improved renal function compared to without hypoxic preconditioning (Weidemann et al. 2008) or expression of dominant negative HIF1A (Tanaka et al. 2005a).

#### HIF-alpha protection against cisplatin-induced renal injury

In contrast to the role of HIFs in cisplatin-induced injury, there is a general consensus that HIFs play a key role in conferring drug resistance through expression of drug transporters, such as ABCB1, detoxification of cisplatin, increased antioxidative capacity, disruption of apoptosis signaling or strengthened ability to repair DNA damage (reviewed in Belisario et al. 2020; Kim and Lee 2017; Shenoy et al. 2017). Indeed, it has been postulated that low HIF expression is decisive in determining the chemosensitivity of testicular cancer (Shenoy et al. 2017) and induction of pseudohypoxia in renal carcinoma cells culminates in augmented chemoresistance and cell motility, which is signaled through HIF-alpha (Liu et al. 2017). In agreement with the notion that HIF upregulation confers protection against cisplatin injury, enhanced HIF1A-induced mitochondrial autophagy (Li et al. 2020), increased HO-1 expression (Bolisetty et al. 2016; Shiraishi et al. 2000) and increased HO-1 activity induced by hemin (Al-Kahtani et al. 2014; Schaaf et al. 2002; Shiraishi et al. 2000) ameliorated oxidative stress, attenuated cisplatin-induced apoptosis of PTCs and increased antioxidative capacity, respectively, in both cell culture and animal models. Co-culture of genetically-engineered HIF1A-expressing stem cells with renal cells (Wang et al. 2014, 2015), administration of conditioned medium from mouse *Hmox1*^+/+^ stem cells (Zarjou et al. 2011) or implantation of EPO-secreting stromal cells (Eliopoulos et al. 2011) to cisplatin-treated mice reduced cisplatin-induced apoptosis and renal injury in a paracrine manner. Furthermore, infusion of rat fetal kidney stem cells ameliorates cisplatin-induced apoptosis with concomitant HIF1A, VEGFA and NOS upregulation, culminating in increased capillary density (Gupta et al. 2015). Finally, recombinant EPO conferred renoprotection against cisplatin-induced injury (Kong et al. 2013; Mohamed et al. 2013; Rjiba-Touati et al. 2011; Zafirov et al. 2008).

In summary, HIFs protect against cisplatin-induced injury to renal cells and fosters development of drug resistance whereas cisplatin toxicity is not HIF-dependent (Table 2). The role of hypoxia in cisplatin nephrotoxicity remains unresolved.

## Therapy of TM nephrotoxicity, controversies, outlook, and conclusions

Chelation of TMs is an obvious therapeutic strategy to prevent further damage, once they have entered the body (reviewed in Flora and Pachauri 2010; Smith 2013). Another important aspect of acute and chronic TM nephrotoxicity is unregulated and overwhelming formation of free radicals (see “Transition metals: environmental presence, exposure, general modes of toxicity in the kidney”), which disrupt physiological ROS signaling, including mitochondrial ROS-dependent activation of the HPHE pathway (see “Contribution of mitochondrial ROS to HPHE signaling” and Fig. 1). In addition, ROS cause detrimental effects to cells through oxidation of proteins, lipids and nucleic acids, which results in cell damage and death. Consequently, application of natural or synthetic antioxidants may be beneficial in the prevention and attenuation of TM-induced renal damage (Flora 2009; Forman and Zhang 2021; Halliwell 2011).

Modulation of the different components of HPHE signaling may also be advocated using pharmacological PHD inhibitors (PHIs) to induce endogenous defense mechanisms, as described with “hypoxic preconditioning” for various causes of AKI and CKD, including TMs (reviewed in Shu et al. 2019; Tanaka 2016; Tiwari and Kapitsinou 2021); see also “General considerations” in “Impact of toxic metal ions on the renal HPHE signaling axis”). PHIs are specific drugs that activate HPHE signaling and were developed to stimulate the production of endogenous EPO in anemia of CKD (Haase 2021). Three oral PHIs (daprodustat, vadadustat, and roxadustat) have advanced to global phase III clinical development (see Table 3 for an overview). On the plus side, PHIs could be useful in the prophylaxis or treatment of TM nephrotoxicity by affecting different mechanisms regulated by HPHE signaling (provided the HPHE axis is intact; see above). They may reduce cell death by inducing HIF activation (Bernhardt et al. 2006a; Conde et al. 2012; Tanaka et al. 2005a; Weidemann et al. 2008), increase HIF-dependent EPO production (for review see Moore and Bellomo 2011), and promote tissue regeneration by HIF activation (Ceradini et al. 2004).

**Table 3:** Clinical trials of orally applied small‑molecule PHD enzyme inhibitors

Drug	Completed Phase	Target	Mechanisms	Results
Roxadustat (FG-4592) Fibrogen AstraZeneca Astellas Pharma	III completed Approved in China (Dhillon 2019)	PHD1 = PHD2 = PHD3 (Haase 2017)	Displacement of 2-OG from PHDs Displacement of HIF-CODD and HIF-NODD from PHD2 (Yeh et al. 2017)	After 6 weeks of treatment in dialysis-dependent (DD) CKD patients dose-dependent Hb ↑. Similar changes of Hb and iron parameters in non-dialysis-dependent (NDD) CKD patients. (e.g. Besarab et al. 2015; Chen et al. 2017; Provenzano et al. 2016)
Daprodustat (GSK-1278863) GlaxoSmithKline	III completed Approved in Japan (Dhillon 2020)	PHD1 = PHD3 ≥ PHD2 (Ariazi et al. 2017)	Displacement of 2-OG from PHDs Displacement of HIF-NODD from PHD2 (Yeh et al. 2017)	Dose-dependent Hb ↑ in DD-CKD patients and NDD-CKD patients. (e.g. Akizawa et al. 2017; Brigandi et al. 2016; Holdstock et al. 2016)
Vadadustat (AKB-6548) Akebia Therapeutics	III completed Approved in Japan (Markham 2020)	PHD3 ≥ PHD1 = PHD2 (Haase 2017)	Displacement of 2-OG from PHDs Displacement of HIF-NODD from PHD2 (Yeh et al. 2017)	Efficient anemia management in DD- and NDD-CKD patients with Hb ↑. (e.g. Haase et al. 2019; Martin et al. 2017; Pergola et al. 2016)

However, regarding therapy of acute versus chronic TM-induced nephrotoxicity a more differentiated approach and a note of caution are necessary. In TM-induced AKI, the literature suggests that PHIs could be useful to increase survival and nephroprotection (reviewed in Li et al. 2021; Shu et al. 2019; Tiwari and Kapitsinou 2021). In contrast, therapeutic application of PHIs for CKD induced by TM ions may be a double-edged sword, first because of the putative proapoptotic (reviewed in Mazure and Pouyssegur 2010; Piret et al. 2002) and pro-fibrotic potential of HPHE activation, due to the interdependence between inflammatory (e.g. nuclear factor kappa B) and HPHE signaling pathways (reviewed in Faivre et al. 2021; Imtiyaz and Simon 2010; Taylor et al. 2016). Second, HIFs induce the expression of proteins involved in Fe/TM transport and homeostasis (e.g. DCYTB, SLC11A2, TF, TFRC, FTH1, HAMP1, MT1A) (reviewed in Shah and Xie 2014; Simpson and McKie 2015). Hence, activation of these two processes could exacerbate chronic renal damage induced by TMs (see also “Transition metals: environmental presence, exposure, general modes of toxicity in the kidney” and “Impact of toxic metal ions on the renal HPHE signaling axis”). Moreover, the carcinogenic potential of metal ions, such as Cd, in the kidney (Hartwig 2013), may be accelerated by activation of HPHE signaling, as exemplified by the proposed role of HIF2A in promoting clear cell renal cell carcinoma (Choueiri et al. 2021; Dufies et al. 2021). On the other hand, PHIs could be beneficial in advanced kidney cancer (reviewed in Burrows and Maxwell 2021). When carefully balancing pros and cons, at the present stage the use of PHIs in humans to slow down or revert CKD induced by nephrotoxic TM ions does not seem recommendable and awaits further developments in the field.

To conclude, acute exposure to relatively high concentrations of TM ions leads to cell death primarily associated with either apoptosis or necrosis, with the latter triggering inflammation and fibrosis. Consequently, these endpoints of acute renal damage may promote disruption of the HPHE pathway, preventing a protective response to damage. Indeed, acute (and chronic) exposure to TMs, such as Fe, Ni, Cd, Cr^6+^ or Pt has been shown to damage the kidney and disrupt HPHE signaling (see “Impact of toxic metal ions on the renal HPHE signaling axis” and Table 2). On the other hand, exposure to low TM ion concentrations may trigger activation of renal HPHE signaling, e.g. by mimicking hypoxia, but also depends on the exposure time. Indeed, Co as well as Fe and Ni (at doses that are not nephrotoxic) do induce HPHE signaling and protect against kidney damage (“preconditioning”; see “General considerations” in “Impact of toxic metal ions on the renal HPHE signaling axis”). Hence, activation of the renal HPHE signaling axis (e.g., by application of pharmacological PHIs under specific circumstances) may delay the onset of acute or chronic renal dysfunction induced by TMs. Strikingly, current knowledge of the field is still very sketchy. For instance, for Ni and Cr^6+^, so far no animal studies on renal HPHE signaling have been performed. Further, for Fe, Ni, Cd and Cr^6+^ no studies investigating HPHE signaling are available in renal cell lines. Clearly, mechanistic elucidation of the role of renal hypoxia–HIF–PHD–EPO signaling in TM nephrotoxicity is necessary before contemplating a rational approach to prevention or therapy.
